# Inhibition of α4β1 Integrin Activity by Small Tellurium Compounds Regulates PD-L1 Expression and Enhances Antitumor Effects

**DOI:** 10.7150/ijbs.95350

**Published:** 2024-08-12

**Authors:** Abigael Chaouat, Yona Kalechman, Ophir Hay, Julia E. Manoim, Tal Lantner, Eitan Niderberg, Hagit Hauschner, Dvora Kenigsbuch Sredni, Tal Cohen, Agata Schlesinger, Ronia Nadler, Mira Barda-Saad, Elad Noy, Michael Albeck, Dan L. Longo, Benjamin Sredni

**Affiliations:** 1 C.A.I.R. Institute, The Safdiè AIDS and Immunology Research Center, The Mina & Everard Goodman Faculty of Life Sciences, Bar-Ilan University, Ramat-Gan, Israel.; 2 Interdisciplinary Dept. Bar Ilan University, Ramat Gan, Israel.; 3 Hospital at Home, Clalit Health Services, Sharon-Shomron District. Department of Geriatrics, Sackler Faculty of Medicine, Tel Aviv University, Tel Aviv, Israel.; 4 The Academic Center of Law and Science, Hod Hasharon, Israel.; 5 Dept. of Chemistry. Bar-Ilan University, Ramat Gan, Israel.; 6 Department of Medicine, Harvard Medical School, Boston, Massachusetts, USA.

**Keywords:** tellurium compounds, immunotherapy, PD-L1, VLA-4, tumor evasion

## Abstract

Various cancer treatment approaches that inhibit the activity of the programmed death-1/programmed death-ligand 1 (PD-1/PD-L1) axis, a key player in tumor immune evasion, have been developed. We show that the immunomodulatory small tellurium complexes AS101 (ammonium trichloro(dioxoethylene-o,o')tellurate) and SAS (octa-O-bis(R,R)-tartarate ditellurane) suppress PD-L1 expression in a variety of human and mouse malignant cells via the modulation of α4β1 very late antigen-*4* (VLA-4) integrin activity. Consequently, the expression of pAkt and its downstream effector pNFκB are inhibited. Additionally, SAS promotes the death of mouse malignant cells by activated syngeneic splenocytes or CD8^+^ T cells, preventing the development of chemoresistance in malignant cells. Moreover, AS101 and SAS may increase, at least in part, chemosensitivity through inhibition of the VLA-4/IL-10/PD-L1 pathway. Additionally, AS101 or SAS treatment of B16/F10 melanoma-bearing mice decreased tumor cell PD-L1 expression, leading to increased CD8^+^ T-cell infiltration into the tumors and tumor shrinkage. Combination treatment with an αPD-1 antibody and either tellurium compound significantly increased the antitumor efficacy of immunotherapy. Overall, VLA-4 integrin signaling is critical for tumor immune evasion and is a potential target for cancer treatment. Finally, AS101 or SAS, biologically active tellurium compounds, can effectively enhance the therapeutic efficacy of αPD-1-based cancer immunotherapy.

## Introduction

The development of immune checkpoint inhibitors (ICIs) for cancer therapy represents one of the most successful approaches in cancer drug discovery in recent years. ICIs serve as the first-line treatment for several types of cancers, such as metastatic melanoma, non-small cell lung cancer, renal cell carcinoma, and bladder cancer. Clinical studies are currently ongoing in other types of cancers, including hematological malignancies 1-5. The programmed death 1/PD ligand 1 (PD-1/PD-L1) axis is a major player in numerous strategies developed to overcome tumor immune evasion. PD-L1 is expressed on various cells, including immune and tumor cells 3, 5. PD-L1 is commonly overexpressed in tumor cells, allowing them to evade the host antitumor immune response 6, survive and accumulate more mutations; this process ultimately results in the cells acquiring resistance to chemotherapy, potentially leading to metastasis and, eventually, patient death 6. Various signaling pathways regulate PD-L1 expression 7-10, including the phosphoinositide 3-kinase (PI_3_K)/Akt 7, 8 and nuclear factor κ-light-chain enhancer of activated B cells (NF-κB) pathways 7, 10.

Most studies have focused on the effects of PD-1/PD-L1 signaling on T cells in the context of cancer 11. In addition to restricting cytotoxic T-cell (CTL) function, the binding of PD-L1 to PD-1 increases the resistance of tumor cells to conventional chemotherapy as well as their proliferation and survival 6. These findings suggest that, in addition to being an effective immune checkpoint blockade strategy, inhibition of the PD-1/PD-L1 axis may be a novel approach to decrease drug resistance in cancer and, therefore, increase the efficacy of conventional chemotherapy.

Many malignant cells express PD-L1. However, only a minor fraction of tumor cells that express PD-L1 respond to therapy 12. To increase treatment efficacy, various combination therapeutic strategies have been suggested, including combination immunotherapy combined with targeted therapy or the addition of chemotherapy to immune checkpoint blockade 13. The combination treatment strategy is regarded as a rational and realistic approach to achieve optimal treatment effects. Accumulating evidence indicates that chemotherapy, radiotherapy, angiogenesis inhibitors, stimulator of interferon genes (STING) agonists, fecal microbiota transplantation (FMT), epigenetic modulators, and other immunomodulators can enhance the therapeutic effects of α-PD-1/PD-L1 immunotherapy by increasing cancer antigen release, enhancing antigen-presenting cell (APC) function, or augmenting effector activity 14-23.

AS101 is a nontoxic tellurium(IV) immunomodulating compound that has been shown to have beneficial effects in diverse preclinical and clinical studies 24-32. AS101 was previously tested in phase II/III clinical trials and has shown efficacy in patients with cancer 24, 27. In a separate study, it is currently being tested in patients with papillomavirus-associated genital warts 33, patients with papillomavirus-associated cervical cancer, and in patients with age-related macular degeneration.

Accumulating evidence suggests that much of the biological activity of AS101 can be directly attributed to its specific chemical interactions with cysteine thiol residues. The tellurium(IV)-thiol chemical bond may lead to a conformational change or disulfide bond formation in a specific protein, possibly resulting in the loss of its biological activity, if the thiol residue is essential for that function 32. Indeed, we demonstrated that the specific redox-modulating activities of AS101 result in a variety of beneficial biological effects including inhibition of interleukin IL-10, resulting in tumor sensitization to chemotherapy 28, 34; and neuroprotection in models of Parkinson's disease 32 and ischemic stroke^35^. We recently described the unique ability of AS101 to regulate cellular VLA-4 activity via redox modulation of vicinal thiols on the exofacial domain of the VLA-4 integrin 29. We demonstrated that the tellurium(IV)-thiol chemical bond formed between AS101 and the α4-chain led to a conformational change in the VLA-4 integrin, preventing its interaction with its specific ligand and consequent signal transduction. We further revealed that tellurium(IV)-mediated inhibition of VLA-4 integrin activity resulted in the conversion of chemotherapy-resistant to chemotherapy-sensitive human myeloid leukemia cells both *in vitro* and *in vivo,* enabling the eradication of residual leukemic cells 29. Moreover, in a xenograft model of acute myeloid leukemia (AML), the combination of chemotherapy with AS101 prolonged survival 29. These specific redox-modulating activities contribute to diverse effects, including improvements in symptoms in experimental autoimmune encephalomyelitis 36, experimental diabetes 37 and colitis model mice 38.

The second-generation tellurium(IV) compound SAS, which we synthesized, possesses the ability to interact with cysteine thiol residues and has similar biological activities to those of AS101. However, this compound has greater stability and solubility in water 30.

On the basis of our previous data showing that inhibition of VLA-4 integrin activity by AS101 29 leads to the inhibition of pAkt and pNFκB expression 29, 39 and on the basis of the role of NFκB in the regulation of PD-L1 expression 12, we designed this study to explore the regulatory effects of these tellurium compounds on PD-L1 expression in diverse human and mouse malignant cells and reveal the physiological role of this effect *in vitro* and *in vivo*.

## Materials and Methods

### Reagents

RPMI-1640 (GIBCO/Thermo Fisher Scientific, MA, USA); L-Glutamine (Biological industries, Bet Haemek, Israel); sodium pyruvate, nonessential amino acids, fibronectin, fetal calf serum (FCS) (Biological industries); VCAM-1 (R&D Biosystems; Minneapolis, MN, USA); BSA, rPD-1 (Sigma, Rehovot. Israel); ARA-C (cytosine β-D-arabinoside) (Sigma); LY294002 (Calbiochem; Dermstadtt, Germany); XTT cell proliferation kit (Biological Industries); anti-pAkt, anti-Akt, anti-pNFκB, and anti-NFκB antibodies (Cell Signaling Technology, MA, USA); anti-CD49d and anti-CD49e antibodies (Serotec, NC, USA); Alexa Fluor 488-conjugated anti-mouse IL-10 antibody (BioLegend, San Diego, CA, USA); lipofectamine (Invitrogen, Carlsbad, CA); PE-conjugated anti-CD8a monoclonal antibody (eBioscience, San Diego, CA, USA); PE-conjugated anti-human PD-L1 antibody (eBioscience); PE-conjugated anti-mouse PD-L1 antibody (Cell Signaling Technology); anti-PD-1 neutralizing antibodies (rat isotype; clone RPMI-14) and isotype control immunoglobulin (rat IgG2a) (Bioxell; Lebanon, In. USA); paclitaxel (Sigma Aldrich); p65-GFP-RelA (Addgene; Watertown, MA, USA); p239-AKT (NIH); VLA-4 shRNA, mouse integrin α4 shRNA, and control plasmid (Santa Cruz Biotechnology; Texas, USA). AS101 and SAS were synthesized at Bar-Ilan University, dissolved in a solution of PBS at a concentration of 150 μg/ml (pH 7.4) and maintained at 4°C.

### Cells

All cell lines were obtained from the American Type Culture Collection. The characteristic DNA profiles of the cells were authenticated via short tandem repeat analysis. The cells were cultured in RPMI-1640 containing 10% fetal calf serum at 37°C with 5% CO_2_ and 95% air. Leukemia cells were obtained from the bone marrow of newly diagnosed AML patients before chemotherapy, following approval by the Institutional Ethics Committee following confirmation of patients' informed consent.

The following cells were used in this study: mouse B16/F10 melanoma cells. (VLA-4 positive); mouse D122 adenocarcinoma alveolar basal epithelial cells (VLA positive); A549 human adenocarcinoma alveolar basal epithelial cells (VLA positive) 40; Wehi-3B mouse myeloid leukemia cells (VLA-4 positive, data not shown); U937 myelomonocytic human leukemia cells (VLA-4 positive); and human AML cells isolated from AML patients (some were VLA-4 positive, some were VLA-4 negative) 29.

### FACS analysis

VLA-4 expression in patient AML cells was determined via fluorescence-activated cell sorting (FACS) after incubation with primary (mouse anti-human Cd49d) and secondary (fluorescein isothiocyanate-conjugated goat anti-mouse immunoglobulin G) antibodies and finally with phycoerythrin-conjugated anti-CD45 antibodies. Blast cells were first identified by CD45/SSC gating in all patients with AML as described by Lacombe and colleagues 41. For intracellular staining, the cells were fixed using 4% paraformaldehyde (PFA), permeabilized using saponin, stained with specific PE-conjugated antibodies and analyzed by FACS. Data analysis was performed using FlowJo software.

### Cell attachment assay

The 96-well plates were coated with 80 μL of VCAM-1 (1 mg/ml) or BSA (2%). The cells were incubated in the wells for 1 hour in the presence or absence of AS101 or SAS. Thereafter, the cells were washed three times. The proportion of attached cells was determined via an XTT (2,3-bis[2-methoxy-4-nitro-S-sulfophenynl]H-tetrazolium-5-carboxanilide inner salt) assay, and the absorbance was read at 450 nm.

### Plasmid transfection

The cells were transfected with p65-GFP-RelA, p65-GFP-RelA, punoAkt or VLA-4 shRNA and their respective control plasmids using Lipofectamine plus reagent (Invitrogen, Carlsbad, CA, USA) according to the manufacturer's instructions. Transfected cells were selected using gentamicin. Stably transfected clones were prepared by single-cell cloning with antibiotics. The transfection efficiency was verified by FACS.

### CD8^+^ T-cell-induced cytotoxicity assay

Malignant cells were cultured on fibronectin-coated plates and cocultured with stimulated syngeneic splenocytes or syngeneic sorted CD8^+^ T cells at a 1:5 ratio. (C57BL/6 splenocytes for B16/F10 and D122 cells and BALB/c splenocytes for Wehi-3B cells). Effector cells were prestimulated with 50 μg of syngeneic malignant cell lysate for 48 h. Stimulated cells were supplemented with rIL-2 (0.1 ng/ml) and lymphocyte growth medium (RPMI 1640 supplemented with 10% FCS, 2 mM L-glutamine, 2 mM sodium pyruvate, 10 mM nonessential amino acids, and 5×10^-5^ 2-mercaptoethanol). The cocultures were further incubated for 48 h. When CD8^+^ cells were used, the sorted CD8^+^ cells were stimulated as described above in the presence of syngeneic adherent spleen cells. The cells were collected and stained with 5 µl of propidium iodide (PI). Malignant cell death was assessed by flow cytometry as the percentage of PI-positive cells among the gated malignant cells.

### IL-10 quantification using ELISA

The cells were cultured on fibronetic (FN)-coated plates and treated for 48 h. The supernatants were collected, and the IL-10 levels were analyzed by ELISA (BioLegend) according to the manufacturer's instructions.

### Fluorescence resonance energy transfer (*FRET*) analysis

FRET analysis was performed using a donor-sensitized acceptor fluorescence technique as previously described 42. Three sets of filters were used: one optimized for donor fluorescence (excitation, 468 nm; emission, 475-505 nm), a second for acceptor fluorescence (excitation, 514 nm; emission, 530 nm longpass; LP), and a third for FRET (excitation, 468 nm; emission, 530 nm LP). FRET was corrected and calculated as previously described 42. Plasmid construction and determination of FRET efficiency are described in detail in 42.

### Animals

Seven- to eight-week-old male C57BL/6 mice were obtained from Harlan Laboratories (Jerusalem, Israel). Animal experiments were performed in accordance with approved institutional protocols. The mice were inoculated subcutaneously with 8×10^4^ B16/F10 melanoma cells. On day 10 (SAS) or 8 (AS101), when the tumors were palpable, the mice were divided into 9 groups (10 mice/group). The mice were treated with different doses of SAS or AS101, as shown in the figures, with or without αPD-1 (250 μg/injection). The experiments included 2 controls**:** PBS and isotype control immunoglobulin. Mice were treated with a single intraperitoneal injection of anti-PD-1 or control antibody on day 11 (SAS experiment) or 9 (AS101 experiment). PBS, SAS or AS101 was administered every other day intraperitoneally starting on day 10 or 8. Tumor length and width were measured every other day, and tumor volume was calculated according to the following formula: tumor volume = width^2^ × length/2. The researchers who measured the tumors were blinded to the treatment groups. In accordance with the ethical committee guidelines, mice whose tumor volume reached 2000 mm^3^ were sacrificed. At the end of the experiment, all the mice were sacrificed, and the tumors were isolated and digested in collagenase IV (0.5 mg/ml). Single cells were stained with anti-PD-L1 antibodies and analyzed by FACS for PD-L1 expression on gated B16/F10 melanoma cells. Furthermore, the cells were stained with anti-CD8 antibodies to evaluate the percentage of CD8^+^ cells infiltrating the tumor.

### Statistics

The results are expressed as mean ± standard error (SE). Differences between groups in *in vitro* experiments and lymphocyte penetration analysis in *in vivo* studies were analyzed via one-way or two-way ANOVA. For tumor volume analysis, two-way ANOVA with multiple comparisons and repeated measures with Bonferroni corrections were applied. The software used for all the statistical analyses was IBM SPSS Statistics 21. p <0.05 was considered statistically significant.

## Results

### SAS and AS101 inhibit PD-L1 protein expression: Role of the VLA-4 integrin

**Figure 1:**
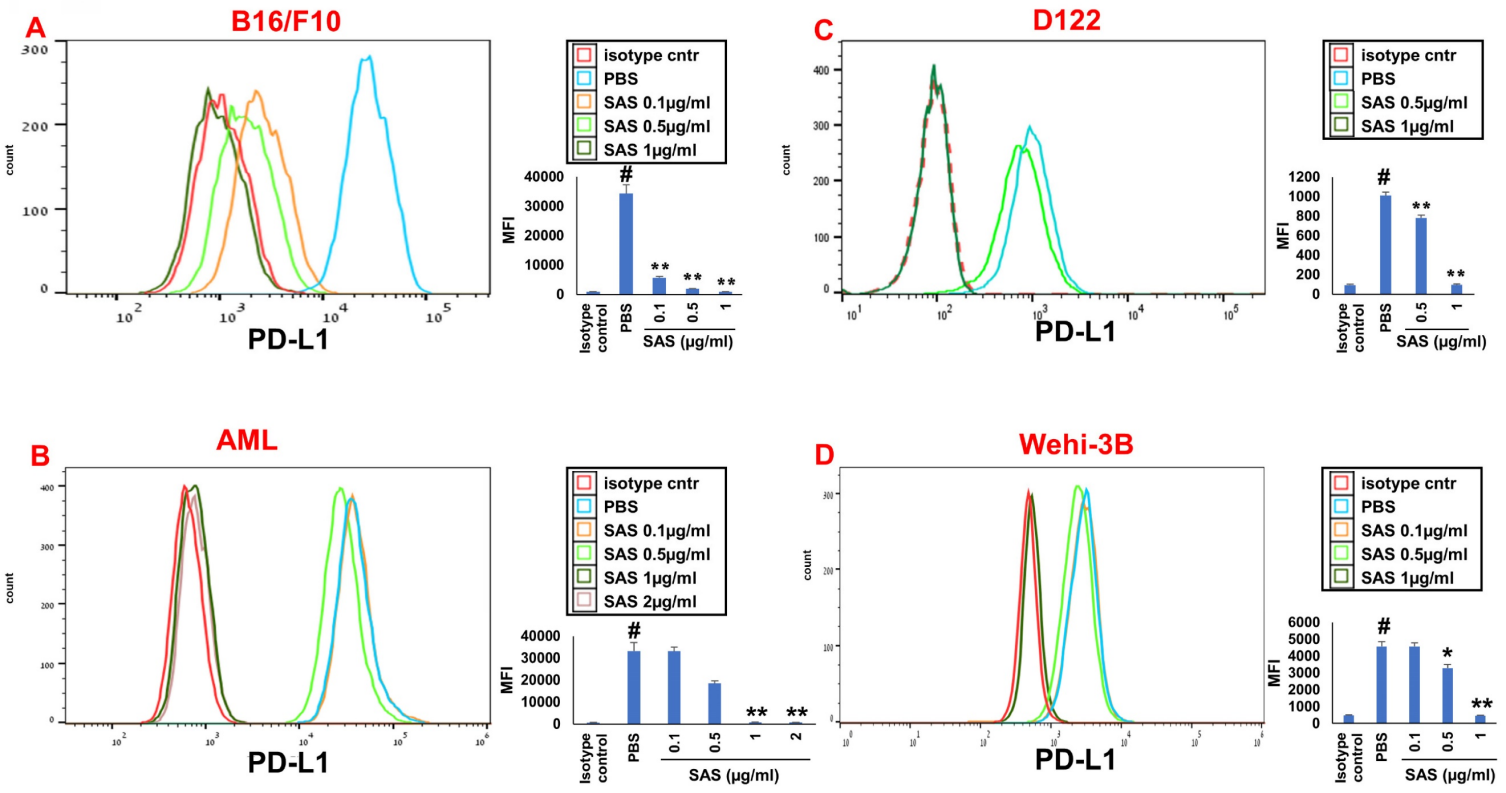
** SAS inhibits PD-L1 protein expression.** Various murine or human malignant cells were cultured on FN-coated plates with or without SAS at various concentrations for 24 hours. The cells tested included (**A**) mouse B16/F10 melanoma cells, (**B**) human VLA-4-positive AML cells isolated from AML patients, (**C**) mouse D122 adenocarcinoma alveolar basal epithelial cells, and (**D**) Wehi-3B mouse myeloid monocytic leukemia cells. The cells were collected and stained with either PE-conjugated anti-mouse or anti-human PD-L1 antibodies or their respective isotype-matched controls. The results show one representative of 3 experiments. Each picture provided is accompanied by quantitative data from flow cytometry analysis. # p<0.001 vs. the isotype control; *p<0.05 vs. the PBS group. **p<0.01 vs. PBS.

**Figure 2:**
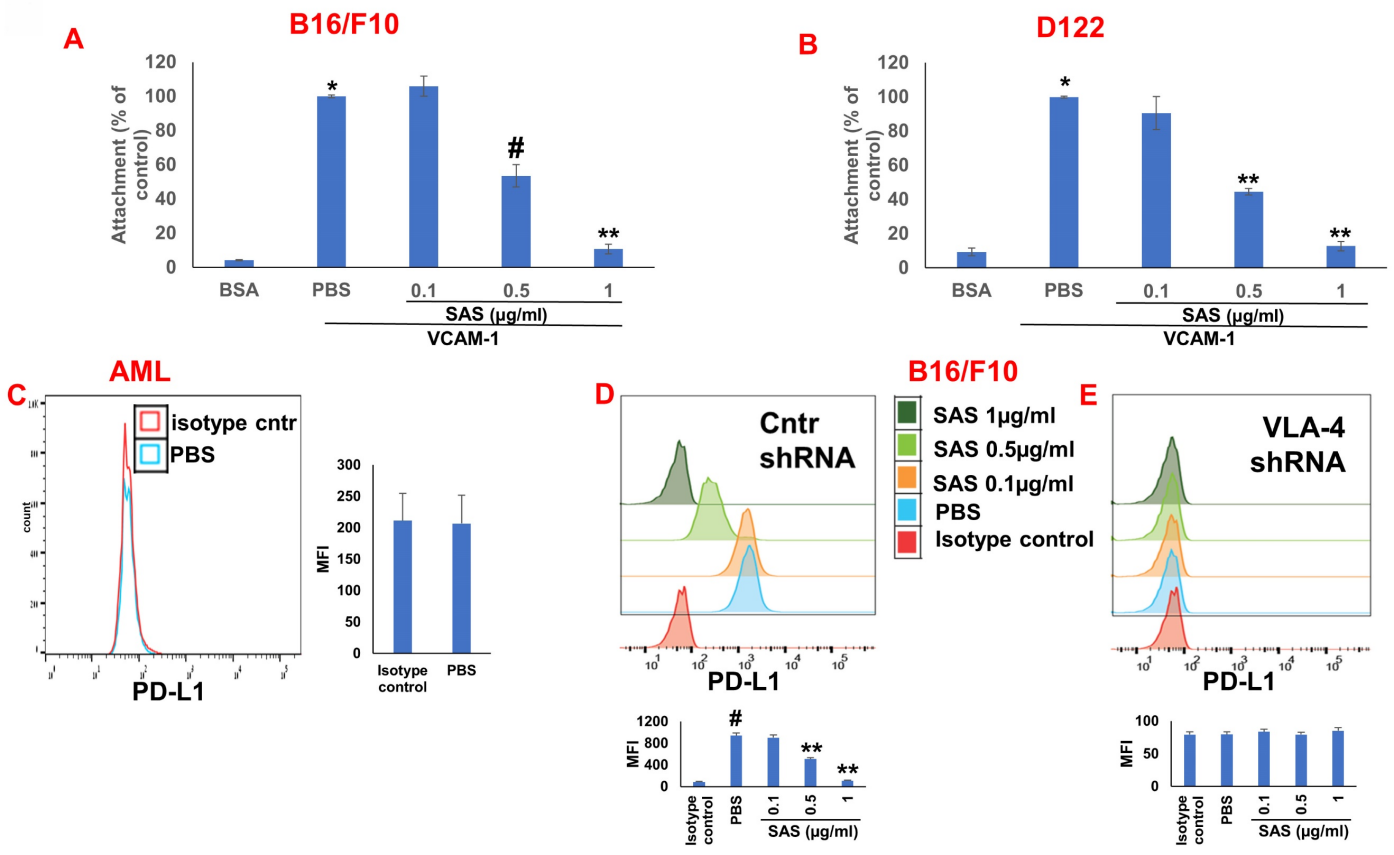
**Role of VLA-4 integrin in PD-L1 expression downregulation by SAS.** (**A**) B16/F10 melanoma cells or (**B**) D122 cells were cultured on vascular cell adhesion molecule-1 (VCAM-1)- or bovine serum albumin (BSA)-coated plates with or without SAS for 1 h. The cells were washed twice**.** The percentage of attached cells, representing VLA-4 (very late antigen-***4***) activity, was determined by the XTT (2,3-bis-(2-methoxy-4-nitro-5-sulfophenyl)-2H-tetrazolium-5-carboxanilide) viability test relative to the control PBS. *p<0.001 vs. BSA; #p<0.01 vs. PBS; **p<0.001 vs. PBS. Significance was calculated via one-way ANOVA. The results are presented as the mean+SE of 3 experiments. (**C**) Human VLA-4-negative AML cells isolated from AML patients were cultured on FN-coated plates for 24 hours. The cells were collected and stained with PE-conjugated anti-mouse PD-L1. (**D**) B16/F10 melanoma cells were transfected with either control or (E) VLA-4 shRNA (short hairpin RNA) and cultured with or without different concentrations of SAS. The cells were collected and stained with either PE-conjugated anti-mouse PD-L1 antibodies or isotype-matched controls. The results show one representative of 3 experiments. Figs 2C, D and E are accompanied by quantitative data from flow cytometry analysis. # p<0.001 vs. the isotype control; *p<0.05 vs. the PBS group. **p<0.01 vs. PBS.

**Figure 3:**
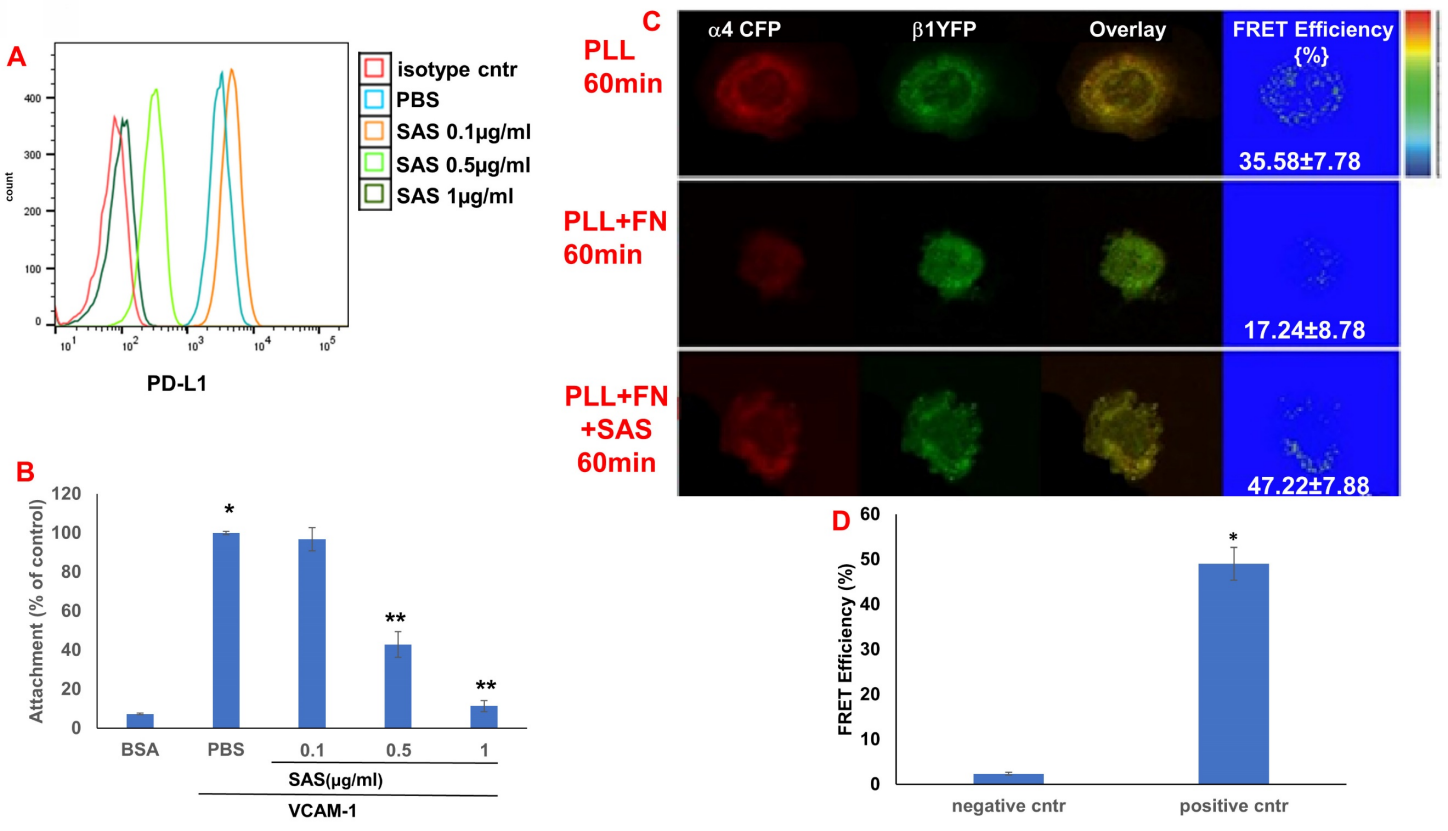
** SAS inhibits both PD-L1 expression and VLA-4 activity in U937 myelomonocytic human leukemic cells.** (**A**) U937 human cells were cultured in the presence or absence of SAS. The cells were collected and stained with either PE-conjugated anti-human PD-L1 antibodies or their respective isotype-matched controls. The results show one representative experiment of 3 performed. (**B**) U937 cells were cultured on VCAM-1- or BSA-coated plates with or without SAS for 1 h. The cells were subsequently washed twice. The percentage of attached cells (representing VLA-4 activity) was determined by the XTT viability test relative to the control PBS. * p<0.001 vs. BSA **p<0.001 vs. PBS. Significance was calculated via one-way ANOVA. The results represent the mean +SE from 3 experiments (**B**). (**C**) The VLA-4 conformational structure was detected by FRET. VLA-4 activation involves the separation of the fluorescently tagged cytoplasmic ends of the α- and β-subunits of the integrin, resulting in a reduction in the FRET signal. U937 cells were transfected with α4-mCFP and β1-mYFP. After 48 hours, the cells were activated and fixed. FRET of the molecular interaction between the cytoplasmic domains of α4 and β1 was determined as described in the Materials and Methods. (**D**) Results for FRET positive controls (cells expressing CFP and YFP encoded on the same plasmid (i.e., maximal FRET obtained in this system) and negative controls (CFP and YFP expressed by different plasmids and undergoing minimal FRET, i.e., only that produced by random colocalizations). *p<0.01 vs. the negative control. Significance was calculated via a two-tailed t test. PLL (poly-L-lysine). FN (Fibronectin).

We first tested the effects of SAS and AS101 on PD-L1 protein expression on the surface of diverse human and mouse malignant cells. As shown in Figure 1, the inhibition of PD-L1 expression by SAS occurred in mouse B16/F10 melanoma cells, human AML cells isolated from AML patients, D122 mouse adenocarcinoma alveolar basal epithelial cells, and Wehi-3B mouse myeloid monocytic leukemia cells. The inhibition was dose-dependent. A total of 1 μg/ml AS101 completely abrogated PD-L1 expression (Fig. 1). AS101 was also shown to inhibit PD-L1 expression in mouse (D122) and human (A549) adenocarcinoma alveolar basal epithelial cells (Fig. S2A and S2B). Each image provided is accompanied by quantitative data from flow cytometry analysis (Fig. 1). We next wished to explore the role of the VLA-4 integrin (the primary target of AS101) in PD-L1 regulation and, specifically, whether inhibition of VLA-4 activity by SAS or AS101 alters PD-L1 expression. For this purpose, we designed several experiments in which VLA-4 activity was modulated by either SAS or AS101, VLA-4 neutralizing antibodies, or shRNAs. Figure 2 shows that SAS disrupts the interaction between VLA-4 and the specific VLA-4 ligand VCAM-1 in B16/F10 melanoma cells (A) and D122 cells (B). This effect is expressed by significant dose-dependent inhibition of the attachment of specific cells to VCAM-1. Similar results were shown with AS101 (Fig. S2C). Thus, VLA-4 activity and PD-L1 expression were both inhibited by SAS and AS101 in malignant cells, as shown in Figure 1 and Figure S2A and B. In addition, in contrast to human AML cell lines, which expressed high levels of VLA-4 and PD-L1 (Fig. 1B), isolated primary AML cells that did not express VLA-4 also lacked PD-L1 expression (Fig. 2C), suggesting a possible association between the expression of these two proteins. All figures showing PD-L1 expression (Fig. 2 C, D and E) are accompanied by quantitative data from flow cytometry analysis. In addition, SAS inhibited PD-L1 expression in human myelomonocytic leukemic U937 cells in a dose-dependent manner (Fig. 3A) and inhibited VLA-4 activity in parallel. This was demonstrated by either interrupting the interaction between VLA-4 on leukemic cells and its specific ligand VCAM-1 (Fig. 3b) or by FRET analysis (Fig. 3C and D). We used the FRET technique to investigate the spatial proximity of the α_4_ and β_1_-cytoplasmic domains in living cells in the presence or absence of FN and SAS, determining the VLA-4 chain configuration and therefore the activation state of the integrin. Figure 3C shows that in the presence of FN, U937 cell cultures presented a decrease in FRET efficiency (17.24±8.78% in the presence of FN vs. 35.58±7.78% in its absence); furthermore, compared with FN treatment alone, treatment of cells with SAS in the presence of FN significantly increased FRET efficiency (47.22±7.88 vs. 17.24±8.78%; *P* < 0.05; controls are presented in Fig. 3D). Neutralizing VLA-4 with anti-VLA-4 antibodies inhibited PD-L1 expression in human A549 cells, and treatment with AS101 had a similar effect (Fig. S2D). Importantly, while B16/F10 melanoma cells transfected with control shRNA (Fig. S1E) expressed PD-L1 (Fig. 2D), which was dose-dependently inhibited by SAS (Fig. 2D), B16/F10 cells in which VLA-4 was neutralized by VLA-4 shRNA (Fig. S1E) did not express PD-L1 (Fig. 2E), and SAS had no effect. These data collectively suggest that in certain malignant cells, active VLA-4 may regulate PD-L1 expression and that its inhibition by either SAS or AS101 leads to the downregulation of PD-L1.

### SAS and AS101 inhibit PD-L1 expression by downregulating pAkt and pNFκB

**Figure 4:**
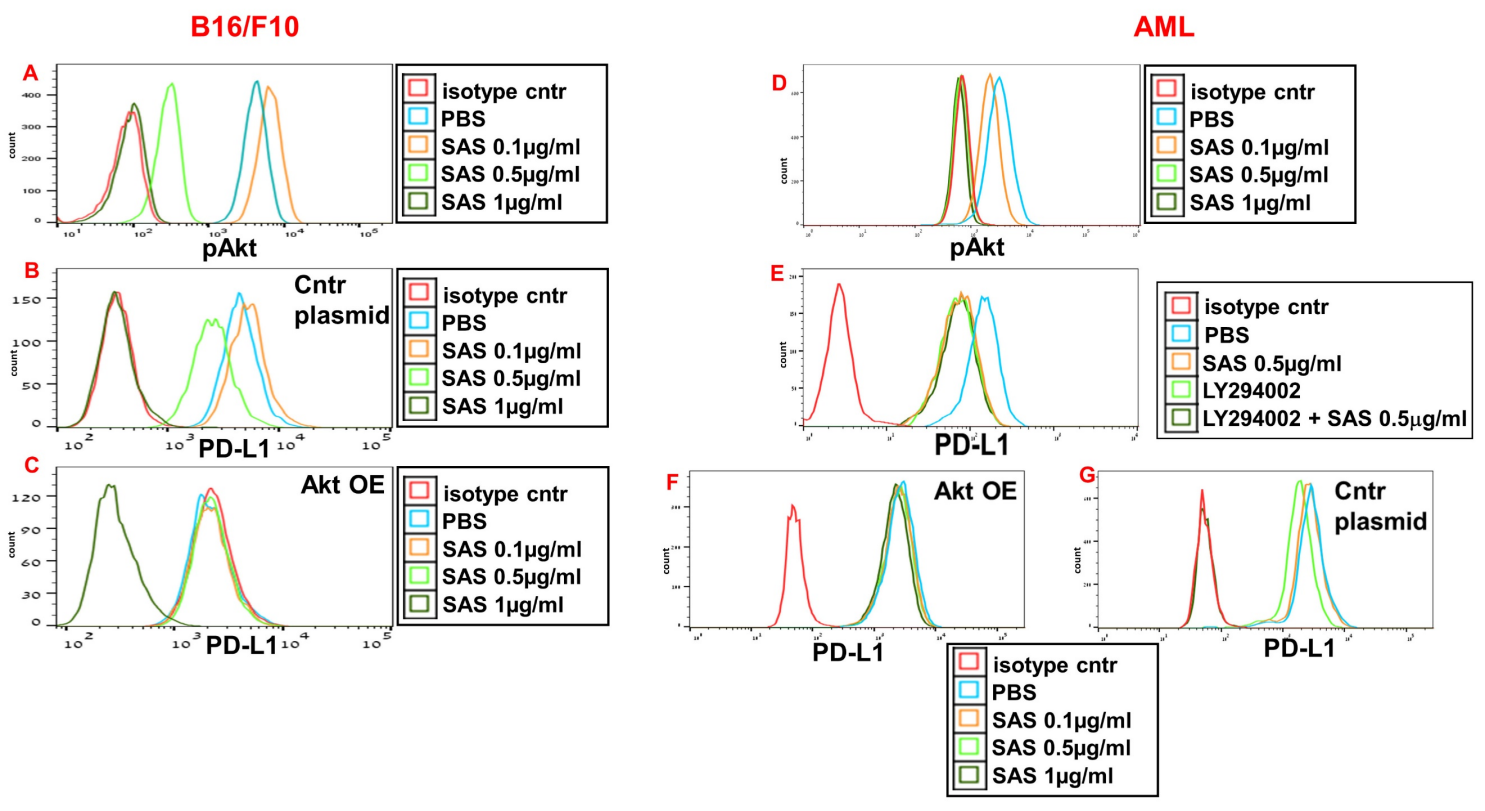
** Inhibition of pAkt expression by SAS and its role in the inhibition of PD-L1 expression by the tellurium compound.** (**A**) Mouse B16/F10 cells, either transfected with (**B**) control plasmid or (**C**) overexpressing (OE) Akt or (**D, E**) human VLA-4-positive AML cells isolated from AML patients or (**F**) AML cells overexpressing (OE) Akt or (**G**) transfected with control plasmid, were cultured on FN-coated plates with or without SAS or with the PI_3_K inhibitor LY294002 (**E**) for 24 hours. The cells were collected, fixed, permeabilized and stained for pAkt (**A, D**) or PD-L1 (**B, C, E-G**). The results show one representative of 3 experiments. Akt (protein kinase B).

AS101 has been previously reported to decrease pAkt and pNFκB expression in certain cells 29, 39, whereas VLA-4 activation is known to increase the protein levels of both Akt and NFκB. We therefore whether the inhibition of pAkt and pNFκB expression by SAS and AS101 plays a role in the regulation of PD-L1 expression. SAS dose-dependently inhibited pAkt expression in B16/F10 melanoma cells (Fig. 4A) and in isolated human AML cells (Fig. 4D), whereas similar results were obtained in A549 human adenocarcinoma alveolar basal epithelial cells treated with AS101 (Fig. S3A). The addition of the PI_3_K inhibitor LY294002 to isolated AML cells inhibited PD-L1 expression in these cells (Fig. 4E), suggesting that pAkt plays a role in PD-L1 expression regulation. Similar results were obtained in A549 cells (data not shown). Moreover, while PD-L1 expression was dose-dependently inhibited by SAS or AS101 in B16/F10 cells (Fig. 4B), AML cells (Fig. 4G) or A549 cells (Fig. S3B) transfected with a control plasmid, this inhibition was abrogated when Akt was overexpressed (Fig. 4C, F and Fig. S3C) (see transfections in Fig. S1A, C and F). These results show that following the inhibition of VLA-4, the primary target of AS101 and SAS, pAkt was downregulated, resulting in the inhibition of PD-L1 expression. Indeed, neutralizing anti-VLA-4 antibodies downregulated pAkt expression in A549 cells to the same extent as AS101 at 0.5 μg/ml (Fig. S2d).

**Figure 5:**
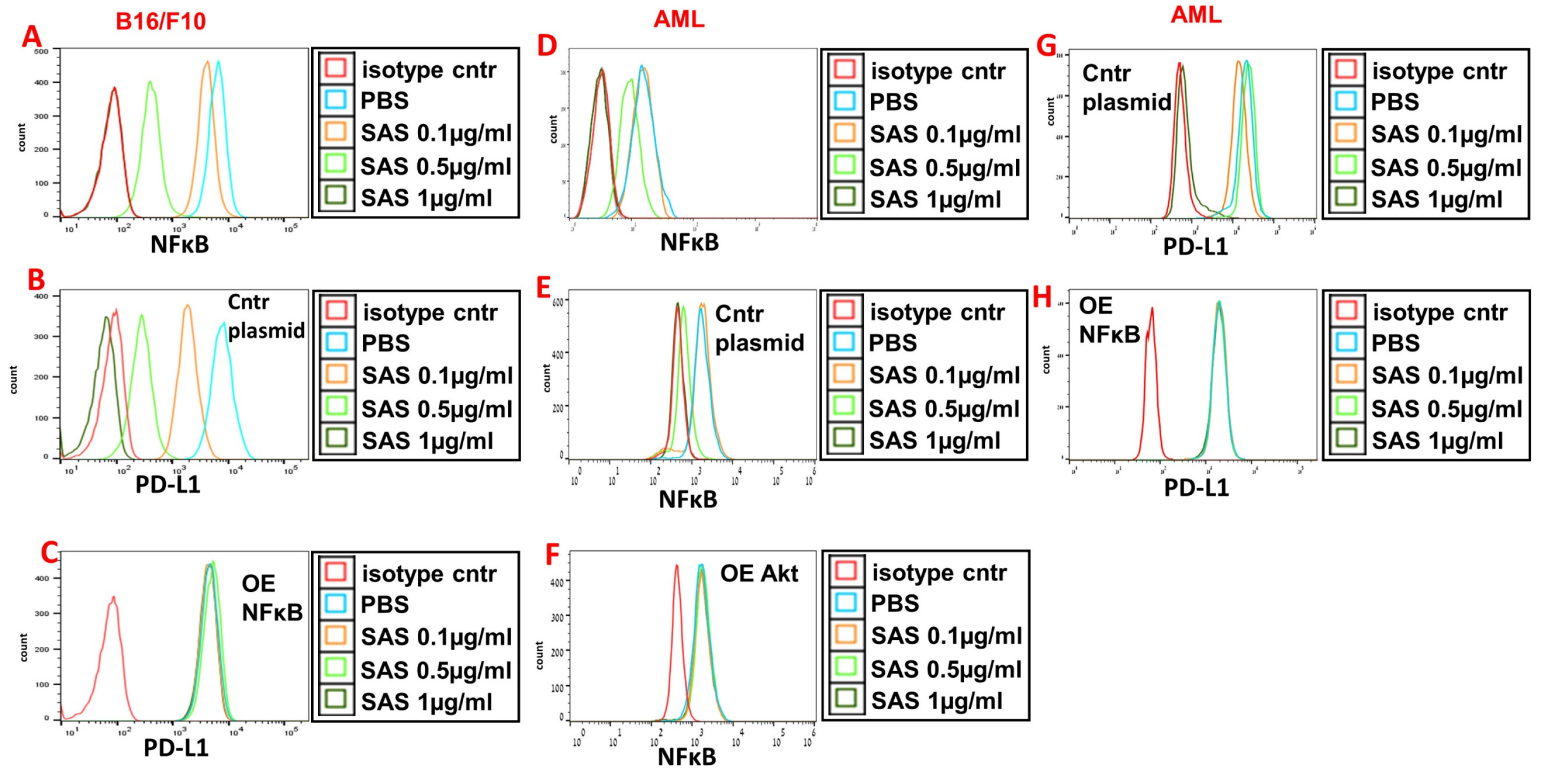
** Inhibition of pNFκB (nuclear factor κ B) expression by SAS and its role in the inhibition of PD-L1 expression by the tellurium compound.** (**A**) Mouse B16/F10 cells either (**B**) were transfected with control plasmid or (**C**) overexpressing (OE) NFκB or (**D**) human VLA-4-positive AML cells isolated from AML patients, either (**E**) transfected with control plasmid or (**F**) overexpressing (OE) Akt or (**H**) overexpressing (OE) NFκB, were cultured on FN-coated plates with or without SAS for 24 hours. The cells were collected, fixed, permeabilized and stained for NFκB (**A, D-F**) or PD-L1 (**B, C, G, H**). The results show one representative of 3 experiments. NFκB (nuclear factor kappa-light-chain-enhancer of activated B cells).

VLA-4 induces the activation of Akt, which then activates the downstream transcription factor NF-κB 29, 43. Indeed, Fig. 5A and D and Figure S3E show the dose-dependent inhibition of pNFκB expression by SAS and AS101 in B16/F10 melanoma cells (A), AML cells (D) and A549 cells (Fig. S3E). The inhibition of pNFκB was dependent on the prior inhibition of pAkt since overexpression of Akt abrogated the decrease in pNFκB levels induced by SAS (Fig. 5F) that was observed in cells transfected with the control plasmid (Fig. 5e). Importantly, the inhibition of PD-L1 expression by SAS or AS101 was dependent on the inhibition of pNFκB since the overexpression of NFκB abrogated this effect (Fig. 5B, C, G, H and Fig. S3E and F) (see transfections in Fig. S1B, D and G). Overall, we suggest that SAS and AS101 inhibit PD-L1 expression on various malignant cells via the inhibition of VLA-4 activity, leading to decreased pAkt/pNFκB levels.

### Associations between VLA-4, IL-10 and PD-L1

**Figure 6:**
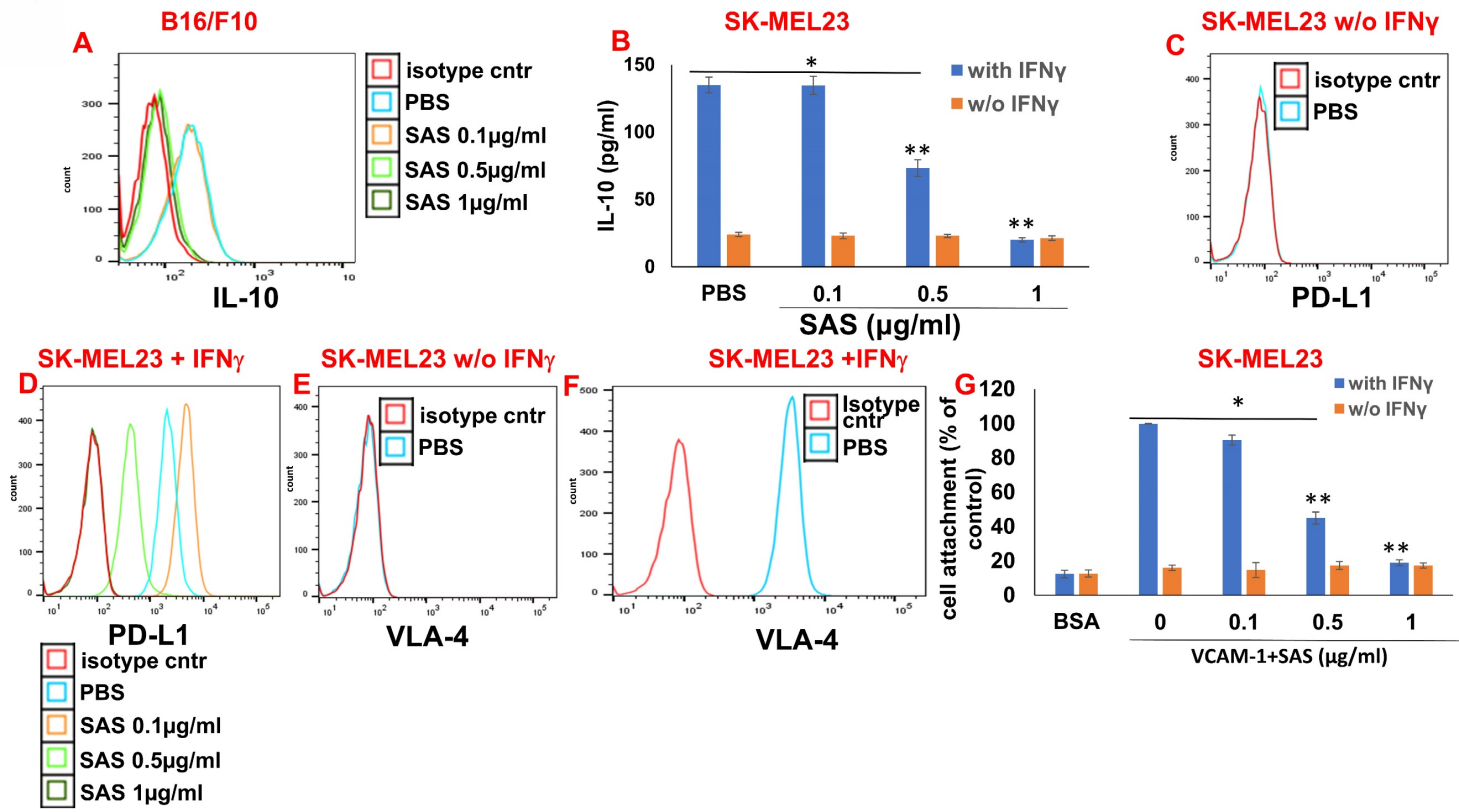
** Associations between VLA-4, IL-10 and PD-L1.** (**A**) B16/F10 cells were cultured on FN-coated plates in the presence or absence of SAS at various concentrations. The cells were collected, fixed, permeabilized and stained with PE-conjugated αIL-10 Abs. (**B**) SK-MEL23 cells were cultured on FN-coated plates in the presence or absence of SAS at various concentrations, with or without IFNγ (2 μg/ml), for 48 h. The supernatants were collected, and IL-10 levels were measured by ELISA. *p<0.0001 vs. without IFNγ; **p<0.001 vs. PBS. Significance was calculated via two-way ANOVA. The results represent the mean+SE of 3 experiments. SK-MEL23 cells without (**C, E**) or with IFNγ (**D, F**) were cultured on FN-coated plates in the presence or absence of SAS at various concentrations for 24 h, after which the cells were collected and stained for PD-L1 (**C, D**) or VLA-4 (**E, F**). All FACS results show one representative of 3 experiments. (**G**) SK-MEL23 cells were cultured in the presence or absence of SAS at various concentrations with or without IFNγ (2 μg/ml) for 24 h. The cells were detached and replated again on VCAM-1- or BSA-coated plates with or without SAS at various concentrations for 1 h. The cells were subsequently washed twice**.** The percentage of attached cells (representing VLA-4 activity) was determined by the XTT viability test relative to the control. *p<0.0001 vs. without IFNγ; **p<0.001 vs. PBS. Significance was calculated via two-way ANOVA. The results represent the mean+SE from 3 experiments. VLA-4 (Very late antigen-4/integrin α4β1). IFNγ (interferon gamma).

IL-10 activates the JAK2/STAT3 pathway, resulting in STAT3-induced PD-L1 expression in various cells 44, 45. We have previously shown that AS101 inhibits IL-10 secretion by a variety of malignant cells 28. This inhibition was VLA-4-dependent 34. We showed that IL-10 inhibition by AS101 resulted in the dephosphorylation of STAT3. Moreover, these effects of AS101 resulted in increased tumor cell sensitivity to chemotherapy 28. Since the regulation of PD-L1 by AS101 and SAS is mediated via the inhibition of VLA-4 activity and since PD-L1 has been reported to induce resistance to chemotherapy in malignant cells 12, we wished to explore the relationships between IL-10 and PD-L1 in the cells studied here. Figure 6A shows a dose-dependent decrease in intracellular IL-10 levels in B16/F10 melanoma cells treated with SAS. Additionally, Figure 6B shows that the human melanoma cell line SK-MEL23 secretes IL-10 only when treated with IFNγ. IL-10 secretion is dose-dependently and significantly decreased by SAS. Moreover, SK-MEL23 cells did not express PD-L1 (Fig. 6C) unless they were treated with IFNγ (Fig. 6D). Under these conditions, PD-L1 expression is dose-dependently decreased by SAS. Importantly, SK-MEL23 cells only slightly expressed the VLA-4 integrin (Fig. 6E) unless they were treated with IFNγ (Fig. 6F). Moreover, in the presence of IFNγ, the VLA-4 integrin is active since cells expressing it attach to the VLA-4-specific ligand VCAM-1 (Fig. 6G). SAS inhibited this activity in a dose-dependent manner, disrupting the interaction between VLA-4 and VCAM-1 (Fig. 6G). Collectively, these results suggest a strong correlation between VLA-4, IL-10 and PD-L1 in malignant cells producing IL-10 and suggest that in these cells, SAS inhibits IL-10 production and secretion, at least partly through the inhibition of VLA-4 activity, leading to decreased PD-L1 expression.

### Inhibition of PD-1/PD-L1-induced chemoresistance and proliferation in malignant cells by SAS

**Figure 7:**
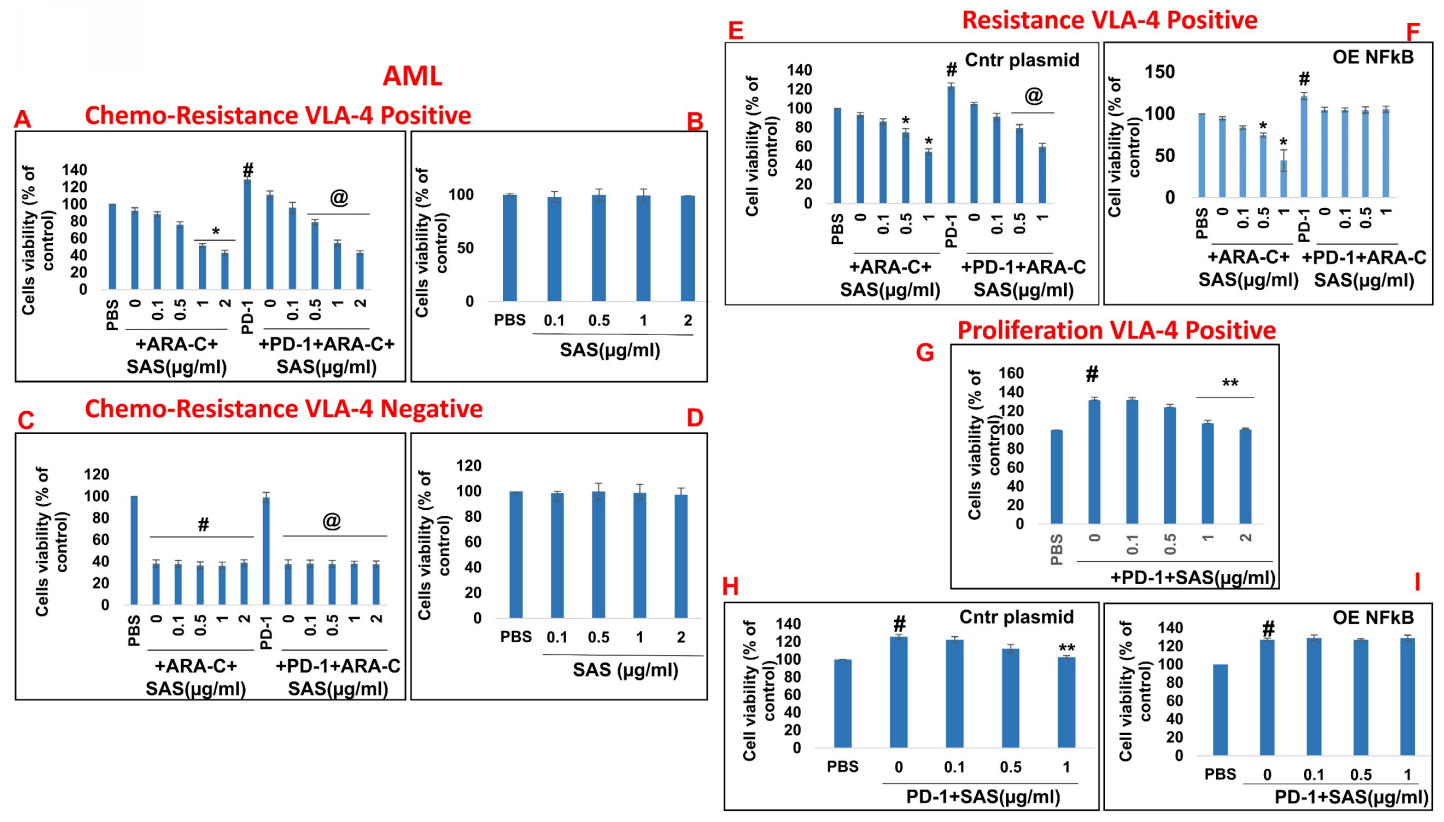
** Inhibition of PD-1/PD-L1-induced malignant cell chemoresistance and proliferation by SAS**: (**A, B, E, F**) Human VLA-4-positive or (**C, D**) VLA-4-negative AML cells isolated from AML patients were cultured on FN-coated plates in the presence (**A**, **C**) or absence (**B, D**) of ARA-C (10^-6^M) or PD-1 (0.2 μg/ml), with or without SAS at the indicated concentrations (**A-D**). Some of the cells (**E**) were either transfected with a control plasmid or (**F**) overexpressing (OE) NFκB. Resistance to chemotherapy (**A, C**) and the dose-dependent effect of SAS alone (**B, D**) on cell viability were tested via the XTT viability test. #p<0.01 vs. PBS; *p<0.05 vs. ARA-C alone; @p<0.01 vs. PD-1. For proliferation assays, cells were cultured on FN-coated plates in the presence or absence of PD-1, with or without SAS at various concentrations (**G-I**). Some of the cells were either transfected with a control plasmid (H) or overexpressing NFκB (I). PD-1-PD-L1-induced proliferation and its inhibition by SAS were tested via XTT cell viability tests. The results represent the mean+SE of 3 experiments; #p<0.05 vs. PBS; **p<0.05 vs. PD-1. Significance was calculated via one-way ANOVA. ARA-C (Arabinosylcytosine).

**Figure 8:**
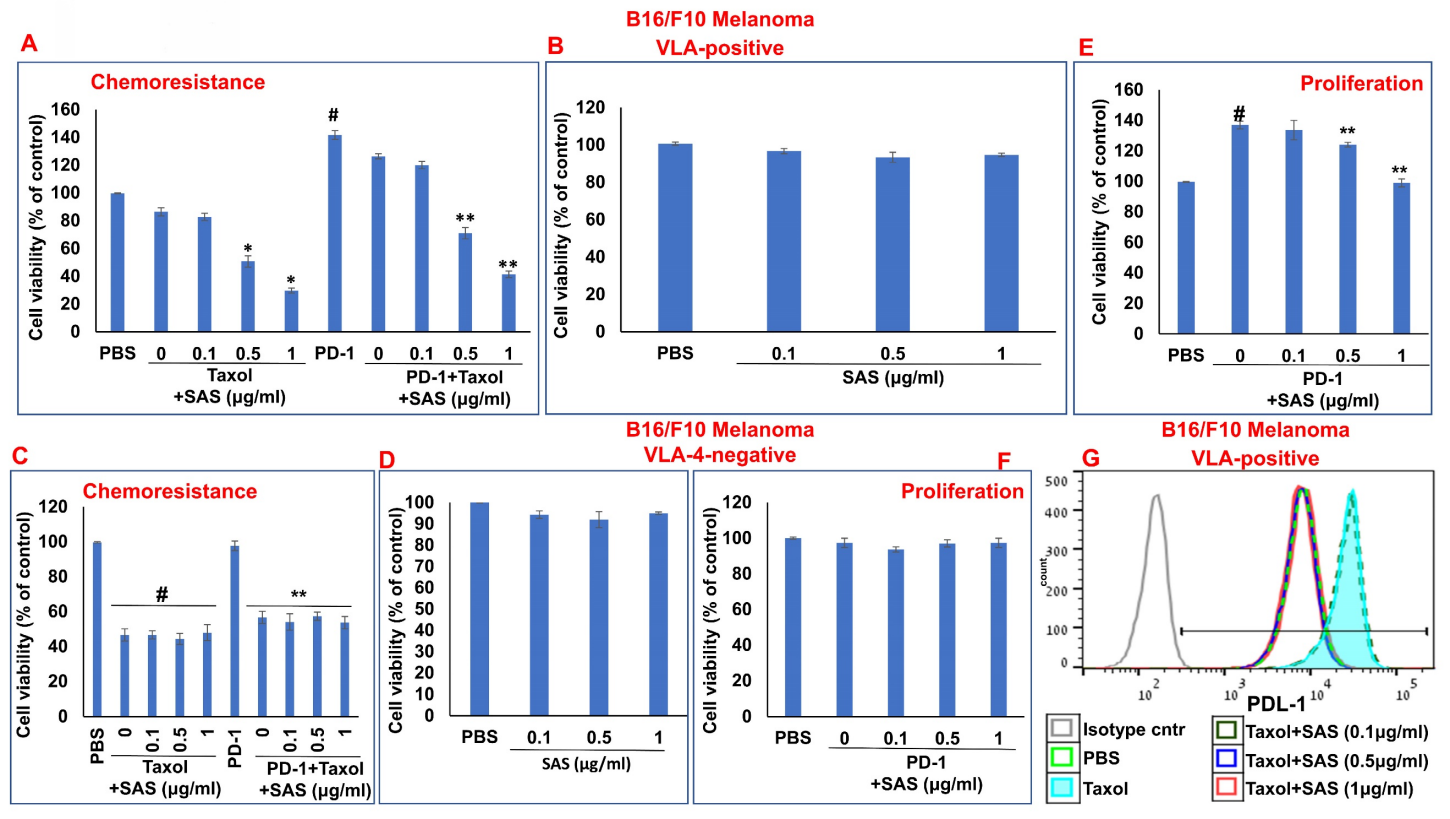
** Inhibition of PD-1/PD-L1-induced B16/F10 cell chemoresistance and proliferation by SAS:** (**A, B**) B16/F10 VLA-4-positive cells or VLA-4-negative cells (**C, D**) were cultured on FN-coated plates in the presence or absence of Taxol (30 nM) or PD-1 (0.2 μg/ml), with or without SAS at various concentrations. Resistance to chemotherapy (**A, C**) and the dose-dependent effect of SAS alone (**B, D**) on cell viability were tested via the XTT cell viability test. #p<0.01 vs. PBS *p<0.01 vs. Taxol alone; **p<0.01 vs. PD-1. PD-1-PD-L1-induced proliferation and its inhibition by SAS (**E, F**) were tested via the XTT viability test. The results represent the mean+SE from 3 experiments; #p<0.01 vs. PBS: **p<0.05 vs. PD-1 (**E, F**). Significance was calculated via one-way ANOVA. (**G**) B16/F10 melanoma cells were cultured on FN-coated plates for 24 h and were thereafter treated with either Taxol alone (30 nM) or Taxol combined with different concentrations of SAS. PD-L1 expression in the cells was analyzed. The data show one representative of 3 experiments.

In addition to PD-L1 in malignant cells interfering with cytotoxic T-cell function, of the binding of PD-L1 to PD-1 increases tumor cell resistance to conventional chemotherapy and increases tumor cell proliferation and survival 6. On the basis of the previously described functions of SAS and AS101 in sensitizing tumor cells to chemotherapy and on the data presented in Figures 1 and S2 showing their inhibitory effects on PD-L1 expression in a variety of malignant cells, we explored the ability of SAS to prevent PD-L1-induced proliferation and drug resistance. Figures 7A and 8A show that isolated AML cells and B16/F10 cells, which are highly VLA-4 positive, are resistant to chemotherapy, whereas treatment with SAS reduces this resistance. SAS alone did not affect cell viability (Fig. 7B and Fig. 8B). of the binding of PD-L1 to PD-1 on both cell types increased cell viability and resistance to chemotherapy, while treatment with SAS significantly sensitized these cells to chemotherapy (Fig. 7A and. 8A). In contrast, VLA-4-negative AML cells (Fig. 7C) or B16/F10 cells (Fig. 8C) responded significantly to chemotherapy, and the response was not enhanced by SAS. In these cells, the binding of PD-L1 to PD-1 did not increase cell viability, probably due to low PD-L1 expression. The overexpression of NFκB in AML cells abrogated the effect of SAS on PD-L1-induced resistance to chemotherapy, potentially by eliminating the ability of SAS to inhibit PD-L1 expression (Fig. 7E and F). Proliferation induced by the binding of PD-L1 to PD-1 was significantly increased in VLA-4-positive AML cells (Fig. 7G) and in B16/F10 cells (Fig. 8E). SAS decreased cell proliferation in both cell types. In contrast, PD-1 did not induce increased proliferation in VLA-4-negative B16/F10 cells, most likely because of PD-L1 deficiency (Fig. 8F). Moreover, overexpression of NFκB prevented the ability of SAS to decrease PD-L1-induced cell proliferation by eliminating the ability of SAS to decrease PD-L1 expression (Fig. 7H and I).

Chemotherapeutic treatments have been shown to increase PD-L1 expression 46, 47, which enhances tumor cell evasion from host immunosurveillance. As shown in Figure 8g, Taxol increased PD-L1 expression in B16/F10 melanoma cells. However, PD-L1 expression was decreased by SAS in a dose-dependent manner. Collectively, these data suggest that SAS prevents PD-L1-induced resistance and proliferation in VLA-4-positive malignant cells, likely through the inhibition of PD-L1 expression. Moreover, the results suggest that treatment with chemotherapy may be feasible when combined with compounds such as SAS that abrogate the chemotherapy-induced increase in PD-L1 expression.

### SAS prevents malignant cell evasion from stimulated syngeneic spleen CD8-positive cells, resulting in malignant cell death

On the basis of these results, we next explored the physiologic effects of downregulating PD-L1 expression in malignant cells via SAS and AS101 both *in vitro* and *in vivo*. We first determined whether treatment of malignant cells with either one of the compounds prevents malignant cell evasion from stimulated syngeneic splenocytes or syngeneic CD8-positive cells, resulting in malignant cell death. For this purpose, we used three cell types: B16/F10 melanoma cells, D122 cells, both originating from C57BL/6 mice, and Wehi-3B cells originating from BALB/c mice. Splenocytes and malignant cells were gated on the basis of their morphology (Fig. S4A, B and C). Table 1 and Figure S5 show that SAS at the highest concentration (1 μg/ml) did not induce malignant cell death in any of the tested cell types. In addition, all the cell types were resistant to stimulated splenocyte- or CD8^+^ T-cell-induced death, probably due to their high PD-L1 expression. However, in the presence of SAS, dose-dependent and significant CTL-induced cell death occurred. Moreover, the overexpression of Akt in B16/F10 cells (Table 1) or NFκB in Wehi-3B cells (Table 1 and Fig. S5) abrogated the ability of SAS to induce CTL-mediated cytotoxicity in these cells. More importantly, compared with both monotherapies, cotreatment of D122 cells with SAS and anti-PD-1 antibodies significantly increased malignant cell death (Table 1). These data collectively suggest that by inhibiting PD-L1 expression, SAS enables CTLs to induce malignant cell death. This effect is significantly potentiated by cotreatment with anti-PD-1 antibodies in an additive manner.

### Treatment of B16/F10 melanoma-bearing mice with SAS or AS101 combined with αPD-1 antibodies results in increased antitumor effects

**Figure 9:**
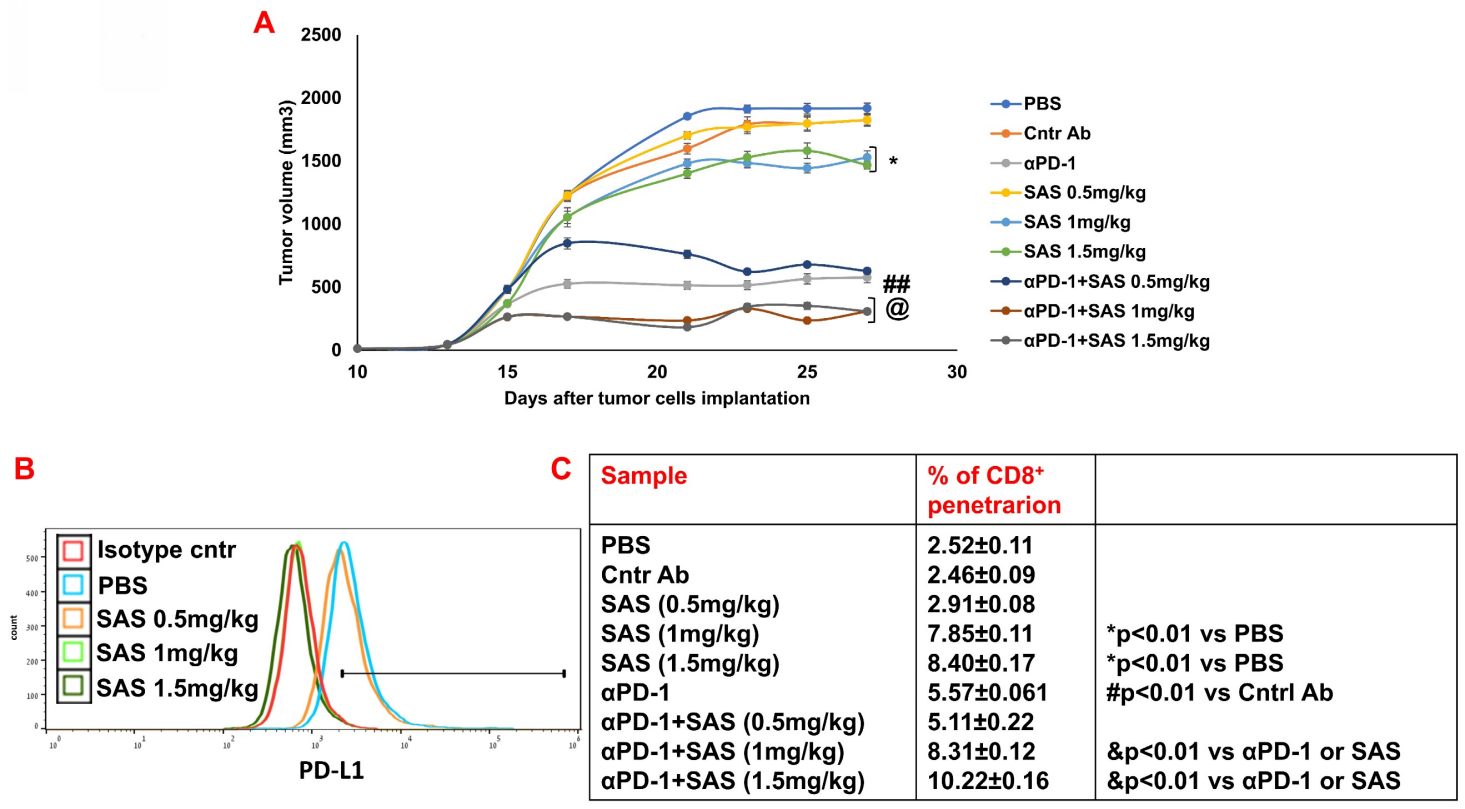
** Treatment of B16 melanoma-bearing mice with SAS reduces tumor volume, decreases tumor cell PD-L1 expression and increases CD8+ cell infiltration into tumors; this effect increases when combined with αPD-1.** (**A**) Male C57BL/6 mice, 7-8 w of age, were inoculated subcutaneously with 8.10^4^ B16 cells/mouse. When the tumors were palpable, the mice were treated intraperitoneally with various concentrations of SAS or PBS every other day in a 0.2 ml volume. Some mice were treated with either αPD-1 antibodies (250 μg/mouse) one day after SAS injection or with an isotype-matched control or SAS+αPD-1 antibodies. The tumor volume was recorded 3-4 times/week (Fig. 9**A**). In accordance with ethical criteria, the mice were sacrificed when the tumor volume reached 2000 mm^3^. N=10/group. *p<0.05 vs. PBS; ##p<0.001 vs. Cntr Ab; @ p<0.01 vs. αPD-1 or vs. SAS 1 or SAS 1.5 mg/kg, respectively. For tumor volume analysis, two-way ANOVA with multiple comparisons and repeated measures with Bonferroni corrections were applied. (**B**) Mice were sacrificed, and their tumors were excised and homogenized to form single cell suspensions. The cells were then stained with an anti-PD-L1 antibody (Fig. 9**B**). PD-L1 expression was determined by FACS analysis of gated tumor cells, as presented in Figure S4D. The results represent data from one of 3 mice/group (Fig. 9**B**). (**C**) Cells were stained with an anti-CD8 antibody. The percentage of infiltrating CD8^+^ cells was determined as the proportion of total cells (malignant + lymphocytes). The data represent the mean+SE of 3 groups of mice. Significance was calculated via one-way ANOVA.

Finally, we performed preclinical experiments with either SAS or AS101 to explore their effects on B16/F10 melanoma-bearing mice either alone or in combination with anti-PD-1. Figure 9A and Figure S6A show that the treatment of tumor-bearing mice with SAS at 1 or 1.5 mg/kg or with AS101 at all concentrations tested significantly decreased tumor volume. Moreover, while αPD-1 was significantly effective in both experiments compared with isotype control antibodies, compared with each treatment alone, cotreatment with αPD-1 and AS101 or with 1 or 1.5 mg/kg SAS further decreased tumor volume. Moreover, the same concentrations of AS101 and SAS, which effectively decreased tumor volume, correlated with decreased PD-L1 expression on tumor cells (Fig. 9B and Fig. S6B). Importantly, these cotreatments with αPD-1 antibodies and the same concentrations of either AS101 or SAS significantly increased CD8^+^ cell infiltration into the tumor compared with that observed in the mice treated with each of these treatments alone (Fig. 9C and Fig. S6C). These data collectively suggest that treatment with SAS or AS101 may decrease tumor volume in B16/F10 melanoma-bearing mice, at least in part, through their ability to decrease PD-L1 expression on tumor cells, enabling more CD8^+^ cells to infiltrate into the tumor and induce tumor cell death. Moreover, the data suggest that cotreatment with αPD-1 immunotherapy and either of the tellurium compounds may further increase CD8^+^ T-cell efficacy in ablating tumor cells, resulting in decreased tumor volume in a synergistic manner.

## Discussion

Inhibition of PD-L1 expression on tumor cells has a dual effect on tumor growth. On the one hand, the host antitumor immune response is enhanced, and on the other hand, resistance to chemotherapy is decreased. In this study, we show that the modulation of VLA-4 activity in a variety of tumor cell lines of either solid tumor or leukemic origin by the tellurium compounds SAS or AS101 reduces PD-L1 expression, preventing malignant cells from evading stimulated syngeneic CD8-positive cells. The release of T cells from PD-1 pathway-mediated inhibition resulted in malignant cell death both *in vitro* and *in vivo*. Furthermore, inhibition of VLA-4 activity led to suppressed PD-L1 expression, resulting in the conversion of PD-1/PD-L1-induced drug-resistant tumor cells to drug-sensitive tumor cells and decreased PD-1/PD-L1-induced cell proliferation. Importantly, combination treatment with these tellurium compounds plus anti-PD-1 antibodies, both *in vitro* and *in vivo*, resulted in an additive effect. To our knowledge, this is the first report showing the regulation of PD-L1 by the VLA-4 integrin.

In the present study, we chose to investigate a variety of malignant cell types to determine if the effects of both tellurium compounds presented here were specific to a certain cell type.

We have previously shown that small tellurium-containing compounds inactivate the VLA-4 integrin on malignant cells via the redox inactivation of adjacent thiols in the exofacial domain of the α4 chain of the integrin 29. This unique mechanism of action affords the compounds relative specificity since these adjacent thiols do not necessarily provide redox-sensitive sites for the regulation of other integrins. Furthermore, AS101 was shown to have an excellent safety profile in patients treated with this compound. This finding suggests that the compound probably has relatively selective primary activity. Thus, inactivation of VLA-4 in tumor cells *in vivo* by AS101 and SAS appears to be direct, leading to a prompt decrease in PD-L1 expression in these cells.

Here, we showed that the inhibition of PD-L1 expression by AS101 and SAS depends on both the inactivation of VLA-4 and its downstream effectors pAkt and pNFκB. Moreover, we demonstrated that the inhibition of each signaling step by both tellurium compounds is dependent on the inhibition of the preceding step; the inhibition of pAkt depends on the suppression of VLA-4, and the inhibition of NFκB depends on the downregulation of pAkt, whereas the inhibition of PD-L1 expression by both compounds depends on the suppression of each signaling step. Furthermore, we demonstrate that the physiological functions of SAS involve PD-L1 expression, which also depend on each of these signaling steps; the ability of SAS to reduce PD-1/PDL-1-induced chemoresistance and proliferation depends on both the suppression of VLA-4 activity and the reduction in pNFκB levels, and its ability to prevent malignant cell evasion from CTL-induced death is dependent on the downregulation of pAkt and pNFκB. Our results are in line with previous reports showing that PI3K/Akt and the transcription factor NFκB can regulate PD-L1 expression 7.

In cancer, PI3K/Akt activation is frequently caused by loss or inactivation of its negative regulator PTEN 48, 49. Loss of other negative regulators of the pathway, such as SHIP or PIB5PA, is also involved in the constitutive activation of PI3K/Akt in some types of cancer 50, 51. While Akt inhibition resulted in decreased PD-L1 expression, its downstream effector, mTOR/S6 kinase, was shown not to mediate Akt-induced PD-L1 expression 52, 53. Although transcriptional upregulation has been shown to affect increased PD-L1 mediated by PI3K/Akt, posttranslational mechanisms are also involved 7. Moreover, Akt activation in colon cancer cells upregulated PD-L1 protein expression without affecting PD-L1 mRNA expression. Overall. the PI3K/Akt pathway likely regulates PD-L1 expression via either transcriptional or posttranscriptional mechanisms in a cell- and tissue-type-dependent manner 54. The activation of AKT reportedly increases HIF-1α protein translation, and it has been reported that the PD-L1 promoter contains an HIF-1α response element 55. NFĸB, a downstream target of Akt, has been shown to regulate PD-L1 56. NF-ĸB is involved in LMP1-induced PD-L1 expression, as the NF-ĸB inhibitor caffeic acid phenethyl ester decreases PD-L1 expression 57. NF-ĸB is also a major mediator of INF-gamma-induced PD-L1 expression 56, 58.

NFκB regulates PD-L1 expression in tumors, either directly at the transcriptional level or via indirect mechanisms. Importantly, different binding sequences for NF-κB have been described on the promoter of the *PD-L1* gene 59-62. PD-L1 expression by cancer cells involves various pathways, all of which activate NFκB, which then binds to the PD-L1 promoter to produce PD-L1. These pathways include the following:

A. Oncogene-related pathways, such as those in which PD-L1 is upregulated by MUC1 63 and EGFR 64 by activating the NFκB pathway. Alternatively, HPV, which controls PD-L1 expression, activates STING to trigger NFκB activation 60. B. Inflammatory cytokine-related pathways. Tumor-infiltrating immune cells can generate several cytokines that control PD-L1 expression. Two cytokines that act through the NFκB pathway are TNFα, which is produced by tumor-associated macrophages (TAMs), and IFN-γ, which is produced by tumor-infiltrating T and natural killer (NK) cells 58.

C. Drug- and stress-related pathways. Different drugs act on NF-κB transcriptional activity 65. The stress response to ultraviolet radiation (UVR) activates NF-κB, thus mediating PD-L1 upregulation 66.

As shown in this study, the regulation of PD-L1 expression by AS101 or SAS requires that both phosphorylated proteins, pAkt and p NFκB, be inhibited by inactivation of the VLA-4 integrin.

Most work on PD-1/PD-L1 signaling in cancer has focused on the effects of PD-L1, particularly those on T cells. However, in addition to interfering with CTL function, the interaction of PD-1 with PD-L1 increases tumor cell resistance to conventional chemotherapy and increases tumor cell proliferation and survival 6. Thus, in addition to effective immune stimulation, inhibition of the PD-1/PD-L1 axis may be a novel approach to decrease drug resistance in cancer and, therefore, increase the efficacy of chemotherapy. Indeed, in the present study, we showed that SAS inhibits PD-1/PD-L1-induced drug resistance and the proliferation of malignant cells. Moreover, we show that the frequently used antitumor drug paclitaxel further increases PD-L1 expression on malignant cells, which promotes drug resistance. This drug-induced effect was found to also be significantly reduced by SAS. Up to 60-70% of patients do not respond to single-agent immune checkpoint blockade therapy. Thus, strategies to reduce PD-L1 expression through strategies other than antibody blockade, which may inhibit chemotherapy-induced augmentation of PD-L1 expression, may improve the outcome of combination chemotherapy and immunotherapy treatment.

We have previously shown that AS101 sensitized tumors to chemotherapy in various tumor-bearing animal models 28. This sensitization to chemotherapy was found to be mediated by the inhibition of the anti-inflammatory cytokine interleukin 10 (IL-10) 28. Moreover, IL-10 inhibition was found to be dependent on inactivation of VLA-4 35. The present study revealed a clear association between VLA-4, IL-10 and PD-L1. The results show that, at least in IL-10-producing malignant cells, both IL-10 secretion and PD-L1 expression depend on VLA-4 activity and that the inhibition of both proteins by the tellurium compounds was mediated by VLA-4 inactivation. It is possible that the inhibition of PD-L1 expression by both tellurium compounds, as shown in the present study, is regulated at least partly by the VLA-4/IL-10 axis. In fact, IL-10 was previously reported to induce PD-L1 expression. In certain T-cell lymphomas, STAT3, which is recruited in response to IL-10 release, bound to the CD274 gene promoter and was required for PD-L1 gene expression 44, 45. Thus, identifying distinct signaling pathways that drive PD-L1 expression may lead to new strategies to mitigate PD-L1 expression.

**Figure 10:**
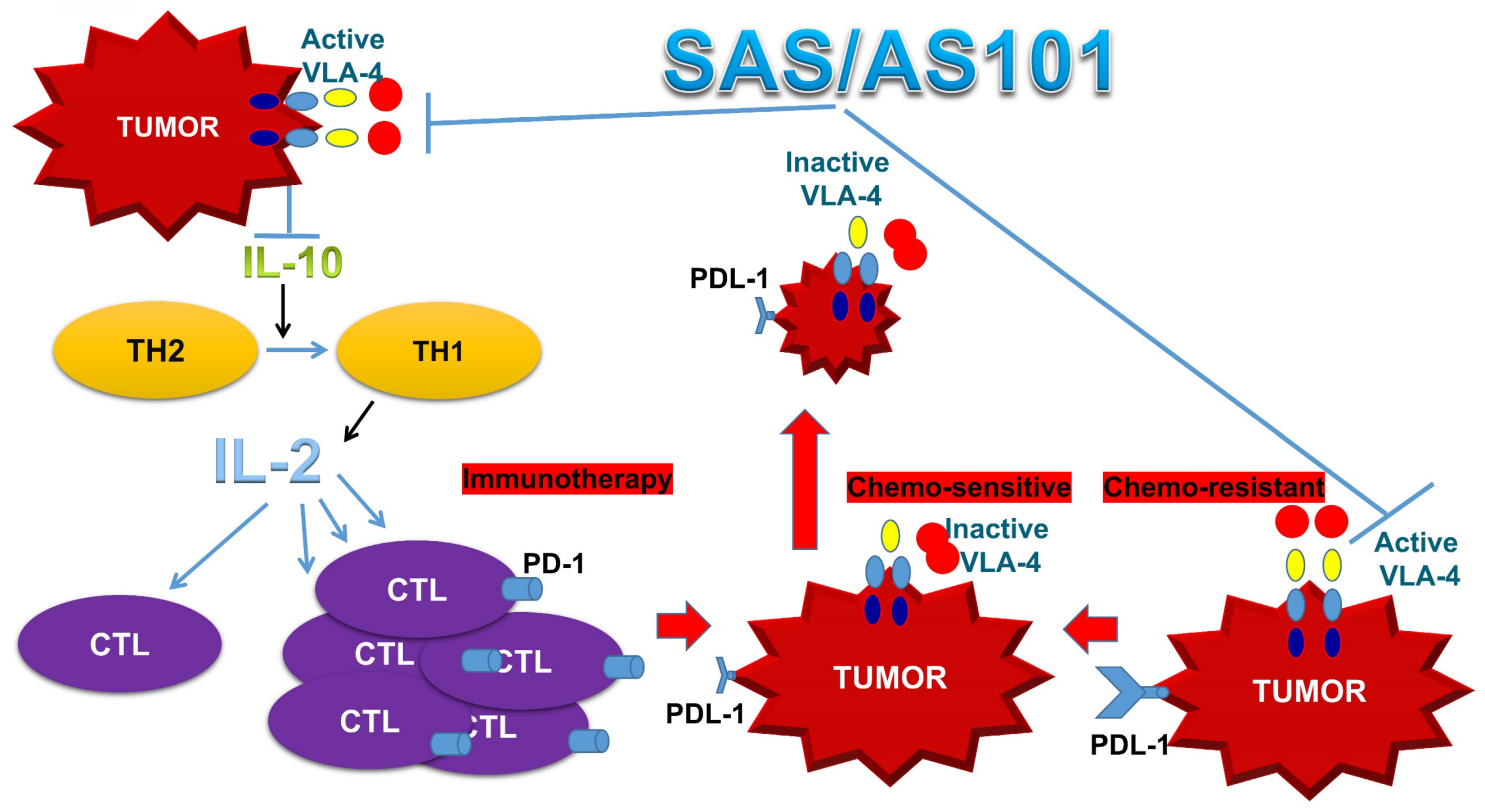
** Schematic of SAS and AS101 enhancing the antitumor effects of immunotherapy by inhibiting the VLA-4/IL-10/PD-L1 axis.** Both tellurium compounds inactivate VLA-4, which is abundantly expressed on many types of malignant cells, resulting in both the inhibition of IL-10 secretion and PD-L1 expression. The inhibition of IL-10 leads to the proliferation and potentiation of CTLs, which, combined with the loss of PD-L1 on tumor cells, can easily infiltrate the tumor and decrease its volume. Moreover, as shown in this *in vitro* study, loss of PD-L1 increases tumor cell sensitivity to chemotherapy. IL-10 (interleukin-10). IL-2 (Interleukin 2). TH1 and TH2 (T helper 1 and 2). CTL (cytotoxic T lymphocyte).

In addition to enhanced chemosensitivity induced by both the inhibition of IL-10 and PD-L1, the suppression of IL-10 by tellurium compounds may lead to a switch in immune responses from Th2 responses to Th1 responses 25, 27. This shift results in the secretion of cytokines such as IL-2 and interferon-gamma, which potentiate the proliferation and enhance the function of CTLs 27. This switch in the immune response and its resulting T-cell potentiation, which are induced by treatment with AS101, has been documented both in preclinical studies involving tumor-bearing mice and in treated cancer patients 27, 67. A summary of the beneficial effects of AS101 and SAS in cancer treatment via inhibition of the VLA-4/IL-10/PD-L1 axis is presented in Figure 10.

Only a fraction of cancer patients respond to checkpoint inhibition therapy. On the basis of our results, we performed *in vivo* studies with B16/F10 melanoma-bearing mice treated with SAS or AS101 alone or in combination with αPD-1 antibodies. This combination produced a significant additive decrease in tumor volume compared with that in mice treated with each compound alone. Importantly, these beneficial effects were associated with decreased tumor PD-L1 expression and increased CD8 positive T-cell infiltration in the tumor.

Among the malignant cells described in the present study, human and mouse adenocarcinoma alveolar basal epithelial cells were included. AS101 and SAS decreased PD-L1 expression in these cells. In randomized placebo-controlled clinical trials previously conducted on unresectable or metastatic non-small cell lung cancer patients (grade IV) who were treated with AS101 combined with chemotherapy (carboplatin and etoposide), unexpected findings were obtained 24. Three out of 20 patients achieved a complete response to the treatment, while no complete response was observed in patients given chemotherapy alone. These findings suggest that in addition to the ability of AS101 to increase tumor sensitivity to chemotherapy, tumor PD-L1 downregulation (PD-L1 expression was not tested at that time) might contribute to enhanced antitumor effects by alleviating tumor-induced immune suppression. The stage is set for testing the combination of AS101 or SAS and anti-PD antibodies, with or without chemotherapy, to increase the number of patients who respond to treatment.

## Supplementary Material

Supplementary figures.

## Figures and Tables

**Table 1:** SAS enhances cytotoxicity by stimulating syngeneic splenocytes or syngeneic CD8-positive cells, resulting in malignant cell death. (A, B) B16/F10 melanoma cells, (C) Wehi-3B cells or (D) D122 cells were cultured on FN-coated cells with or without (Ai, C, D) stimulated syngeneic splenocytes or (Aii, B) sorted spleen CD8^+^ cells. Stimulation was performed with 50 μg of syngeneic malignant cell lysate for 48 h. The stimulated cells were supplemented with rIL-2 (0.1 ng/ml) and lymphocyte growth medium. The cocultures were treated with or without SAS and further incubated for 48 h. When CD8^+^ cells were used, the sorted CD8^+^ cells were stimulated as described above in the presence of syngeneic adherent spleen cells. The cells were collected and stained with 5 µl of PI. The death of target malignant cells was assessed by flow cytometry as the percentage of PI-positive cells among the gated malignant cells. The results represent the mean±SE from 3 experiments. *p<0.001 vs. malignant cells+ splenocytes or with CD8^+^ cells; #p<0.01 vs. malignant cells+splenocytes; **p<0.001 vs. malignant cells+ splenocytes+αPD-1. Significance was calculated via one-way ANOVA. Akt (protein kinase B). NFκB (nuclear factor kappa-light-chain-enhancer of activated B cells).

Aii) % Death of B16/F10 cells w/wo sorted spl CD8^+^ cells		Ai) % **Death of B16/F10 cells w/wo splenocytes**	A) Samples			
9.00±1.25		8.25±2.81	**Malignant cells alone+PBS**			
12.86±0.92		9.44±3.16	**Malignant cells alone+SAS 1** µ**g/ml**			
15.06±1.35		10.61±4.95	**Malignant cells+splenocytes+PBS**			
15.23±2.97		17.30±1.68	**Malignant cells+splenocytes+SAS 0.1** µ**g/ml**			
55.83±5.52*		50.26±7.71*	**Malignant cells+splenocytes+SAS 0.5** µ**g/ml**			
63.03±5.52*		68.86±7.33*	**Malignant cells+splenocytes+SAS 1** µ**g/ml**			

% Death of Wehi-3B cells w/wo splenocytes	C) Samples			% Death of B16/F10 cells w/wo sorted spl CD8^+^ cells	B) Samples	
8.46±0.4		Cntr malignant cells alone+PBS		11.87±3.70		Cntr malignant cells alone+PBS
7.6±0.46		Cntr malignant cells alone+SAS 1 µg/ml		11.25±4.00		Cntr malignant cells alone+SAS 1 µg/ml
11.03±2.38		Cntr malignant cells+splenocytes+PBS		14.53±2.90		Cntr malignant cells+spl CD8^+^
19.1±1.28**		Cntr malignant cells+splenocytes+SAS 0.1 µg/ml		12.60±3.24		Cntr malignant cells+spl CD8^+^+ SAS 0.1 µg/ml
45.5±1.70*		Cntr malignant cells+splenocytes+SAS 0.5 µg/ml		37.13±4.53*		Cntr malignant cells+spl CD8^+^+ SAS 0.5 µg/ml
62.36±2.42*		Cntr malignant cells+splenocytes+SAS 1 µg/ml		52.43±3.75*		Cntr malignant cells+spl CD8^+^+ SAS 1 µg/ml
7.23±1.03		NFκB OE Malignant cells alone +PBS		11.24±3.05		Akt OE Malignant cells alone +PBS
6.89±2.10		NFκB OE Malignant cells+splenocytes		10.66±0.28		Akt OE Malignant cells+spl CD8^+^
7.27±0.83		NFκB OE Malignant cells+SAS 1 µg/ml		11.15±2.76		Akt OE Malignant cells+SAS 1 µg/ml
6.98±0.87		NFκB OE Malignant cells+splenocytes + SAS 0.1 µg/ml		10.40±2.05		Akt OE Malignant cells+spl CD8^+^+ SAS 0.1 µg/ml
8.04±0.24		NFκB OE Malignant cells+splenocytes+ SAS 0.5 µg/ml		11.36±2.48		Akt OE Malignant cells+spl CD8^+^+ SAS 0.5 µg/ml
7.55±1.19		NFκB OE Malignant cells+splenocytes+ SAS 1 µg/ml		10.56±3.01		Akt OE Malignant cells+spl CD8^+^+ SAS 1 µg/ml

% Death of D122 cells w/wo splenocytes		D) Samples				
5.85±0.35		Malignant cells alone+PBS				
6.04±0.78		Malignant cells alone+SAS 1 µg/ml				
5.40±0.44		Malignant cells alone+**α**PD-1				
4.83±0.90		Malignant cells alone+**α**PD-1+SAS 1 µg/ml				
5.12±0.56		Malignant cells+splenocytes+PBS				
5.26±0.55		Malignant cells+splenocytes+ SAS 0.1 µg/ml				
31.73±2.2*		Malignant cells+splenocytes+ SAS 0.3 µg/ml				
69.8±3.05*		Malignant cells+splenocytes+ SAS 0.5 µg/ml				
86.9±3.3*		Malignant cells+splenocytes+ SAS 1 µg/ml				
5.22±0.62		Malignant cells+splenocytes+ Isotype cntr				
48.4±2.1**		Malignant cells+splenocytes+ **α**PD-1				
83.15±2.85**		Malignant cells+splenocytes+ **α**PD-1+ SAS 0.3 µg/ml				
82.66±1.71**		Malignant cells+splenocytes+ **α**PD-1+ SAS 0.5 µg/ml				
84.05±3.10**		Malignant cells+splenocytes+ **α**PD-1+ SAS 1 µg/ml				
						
