# Supplementary material for: Inhibition of α4β1 Integrin Activity by Small Tellurium Compounds Regulates PD-L1 Expression and Enhances Antitumor Effects
Source: Int J Biol Sci. 2024 Aug 12;20(11):4407–23. doi: 10.7150/ijbs.95350 (PMC11380454; doi:10.7150/ijbs.95350)
Supplement: Supplementary file 1 — Supplementary figures. [file ijbsv20p4407s1.pdf]

Supplementary Figure Legends

Fig. S1. (**A, C, F**) Overexpression of Akt and (**B, D, G**) NFκB in AML, B16/F10 or A549 cells, respectively. (**E)** Decreased VLA-4 expression by VLA-4 shRNA in B16/F10 cells.

Fig. S2. AS101 inhibits PD-L1 protein expression. (**A**) D122 mouse and (**B**) A549 human adenocarcinoma alveolar basal epithelial cells were cultured on FN-coated plates with or without AS101 at various concentrations or (**D**) with either AS101 (0.5 µg/ml) or αVLA-4 neutralizing Abs (10 µM) alone or in combination for 24 hours. The cells were collected and stained with either PE-conjugated anti-mouse or anti-human PD-L1 Abs or with their isotype-matched control. The results show one representative experiment of 3 performed. (**C**) A549 cells were cultured on VCAM-1- or BSA-coated plates with or without AS101 for 1 h. The cells were subsequently washed twice**.** The percentage of attached cells (representing VLA-4 activity) was determined by the XTT viability test relative to the control PBS. #<0.001 vs. BSA; **p<0.001 vs. PBS. Significance was calculated via one-way ANOVA.

Fig. S3. Inhibition of pAkt and NFκB expression by AS101 in A549 cells and its role in the inhibition of PD-L1 expression by the compound: (**A, D**) A549 cells (**B, E**) either transfected with control plasmid or (**C**) overexpressing Akt or (**F**) NFκB were cultured on FN-coated plates with or without AS101 for 24 hours. The cells were collected, fixed, permeabilized and stained for pAkt (**A**) or NFκB (**D**). **(B-F)** Alternatively, the cells were collected and stained for PD-L1. The results show one representative experiment of 3 performed.

Fig. S4. Gating strategy: (**A, B, C**) Side scatter (SSC)/forward scatter (FSC) plots of malignant cells alone, splenocytes alone, or both combined in experiments in which (**A**) B16/F10 melanoma cells, (**B**) D122 cells or (**C**) Wehi3B cells were used. (**D**) SSC/FSC plots of isolated cells from excised tumors from either SAS- or AS101-treated mice, showing gated tumor cells or splenocytes.

Fig. S5. SAS prevents the evasion of Wehi-3B malignant cells from stimulated syngeneic splenocytes, resulting in malignant cell death. (**A-H**) Wehi-3B cells were either transfected with a control plasmid or (**I-L**) overexpressing NFκB. were cultured on FN-coated cells with or without stimulated syngeneic splenocytes from BALB/c mice. Stimulation was performed with 50 µg of syngeneic malignant cell lysate for 48 h. The stimulated cells were supplemented with rIL-2 (0.1 ng/ml) and lymphocyte growth medium. The

cocultures were supplemented with or without SAS and further incubated for 48 h. The cells were collected and stained with PI. The percentage of malignant cell death was determined via FACS analysis as the percentage of PI-positive gated malignant cells. The data represent one of three experiments.

Fig S6. Treatment of B16 melanoma-bearing mice with AS101 reduces the tumor volume, decreases tumor cell PD-L1 expression, and increases $CD8^+$ cell infiltration into the tumor; this effect is enhanced by combined with αPD-1 treatment. (**A**) Male C57BL/6 mice, 7–8 w of age, were inoculated subcutaneously with 8.10^4 B16 cells/mouse. When the tumors were palpable, the mice were intraperitoneally every other day with various concentrations of AS101 or PBS until the end of the experiment in a 0.2 ml volume. The indicated mice were treated with αPD-1 Ab (250 µg/mouse), isotype-matched control, or AS101 and αPD-1 Ab at 1 day after AS101 injection. The tumor volume was recorded 3–4 times/week. In accordance with ethical guidelines, the mice were sacrificed when the tumor volume reached 2000 mm^3. N=10/group. *$p<0.05$ vs. PBS; **$p<0.01$ vs. Cntr Ab; # $p<0.01$ vs. αPD-1, AS101 1, or AS101 1.5 mg/kg. For tumor volume analysis, two-way ANOVA with repeated measures with Bonferroni corrections was used for multiple comparisons. (**B**) PD-L1 expression. The mice were sacrificed, and their tumors were excised and homogenized to form single cell suspensions. The cells were stained with an anti-PD-L1 antibody. PD-L1 expression was determined by FACS analysis of gated tumor cells, as presented in Figure S4e. The results represent the mean±SE of 3 groups of mice.. (**C**) Cells were stained with an anti-CD8 antibody. The percentage of infiltrating $CD8^+$ cells was determined as the proportion of total cells (malignant + lymphocytes). The data represent the mean±SE of 3 groups of mice. Significance was calculated via one-way ANOVA.

Supplementary Figures

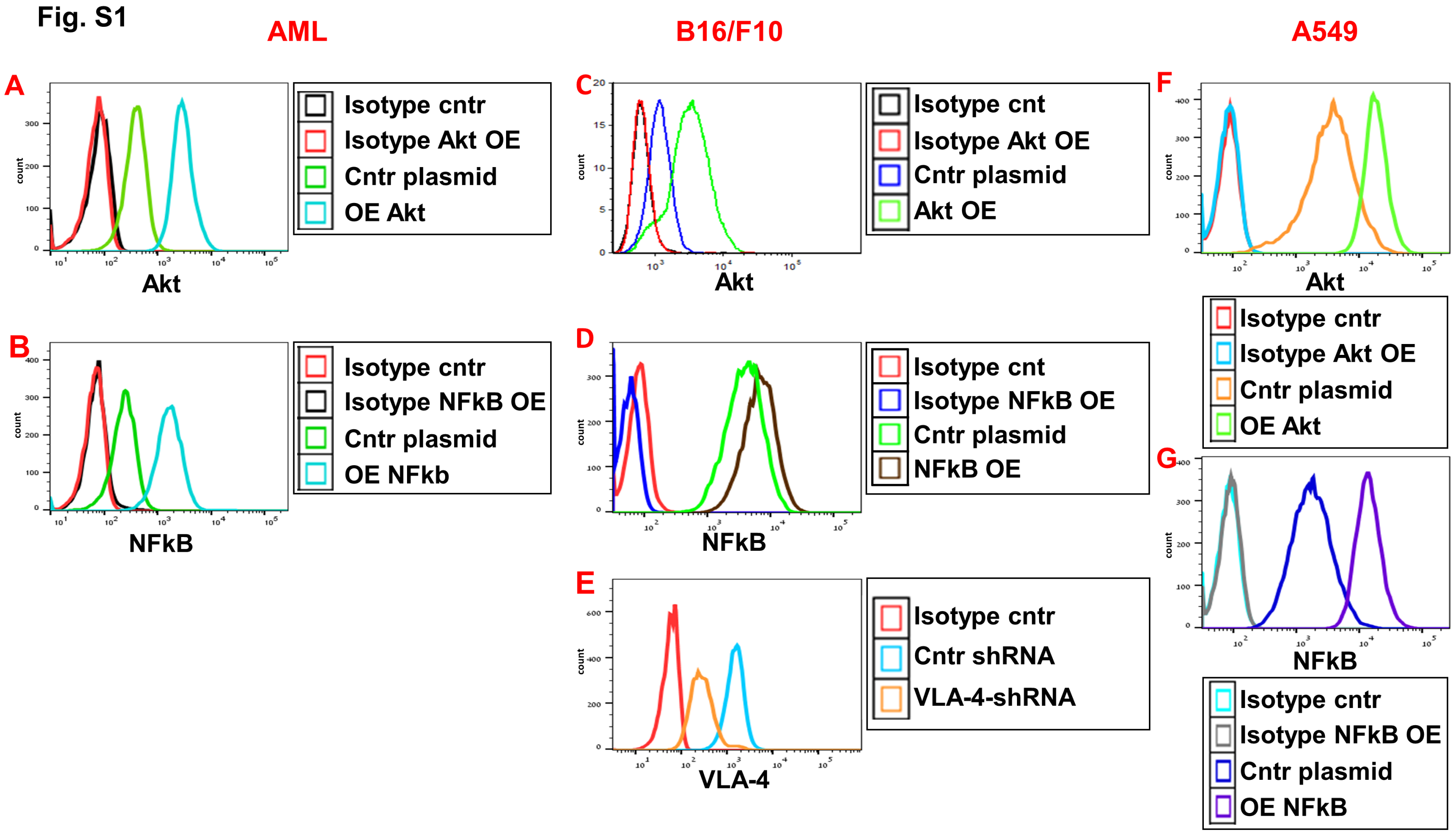

Fig. S2

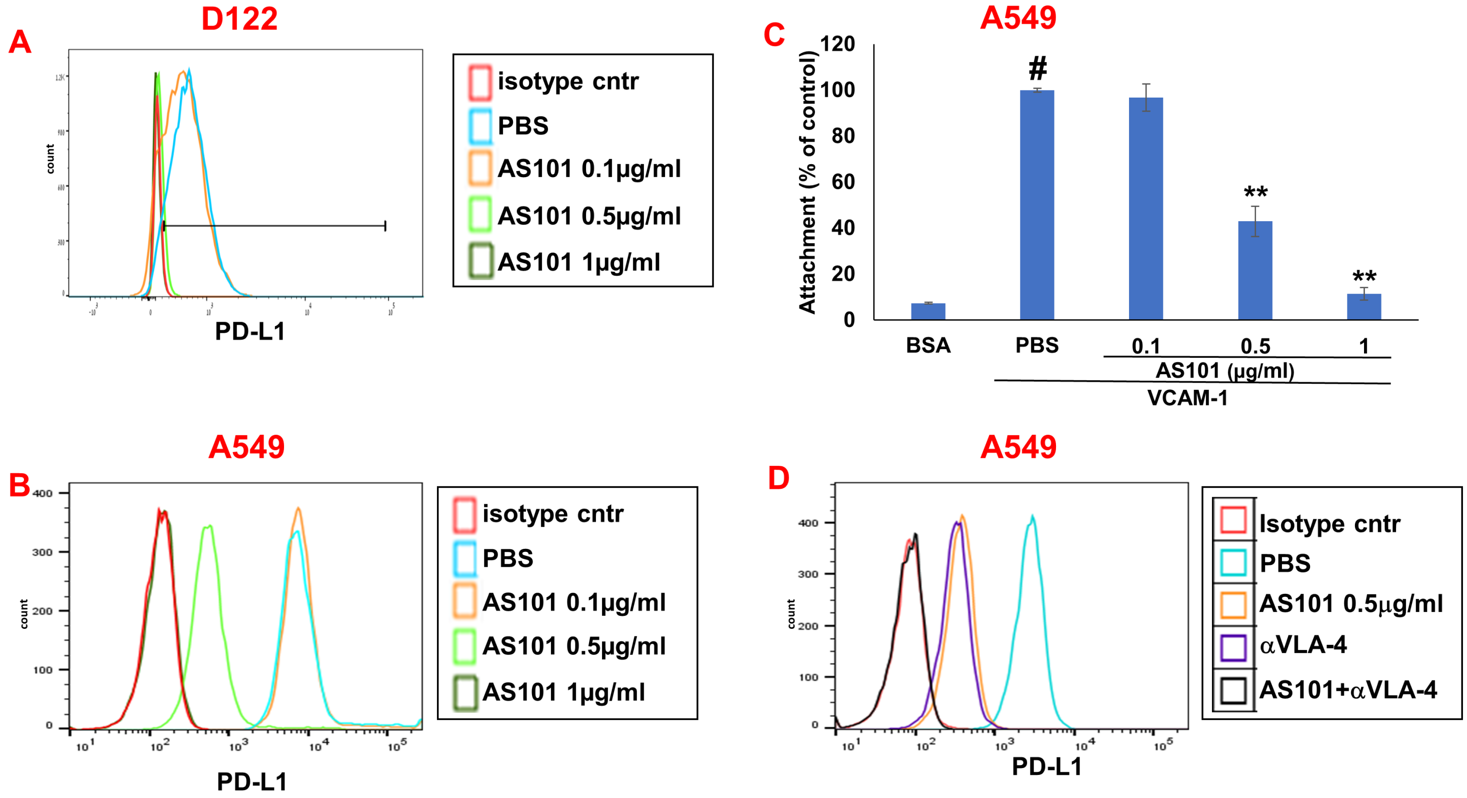
A
D122
count
PD-L1
isotype cntr
PBS
AS101 0.1μg/ml
AS101 0.5μg/ml
AS101 1μg/ml
C
A549
Attachment (% of control)
120
100
80
60
40
20
0
#
**
**
BSA
PBS
0.1
0.5
1
AS101 (μg/ml)
VCAM-1
B
A549
400
300
200
100
0
count
PD-L1
isotype cntr
PBS
AS101 0.1μg/ml
AS101 0.5μg/ml
AS101 1μg/ml
D
A549
400
300
200
100
0
count
PD-L1
Isotype cntr
PBS
AS101 0.5μg/ml
αVLA-4
AS101+αVLA-4

Fig. S3

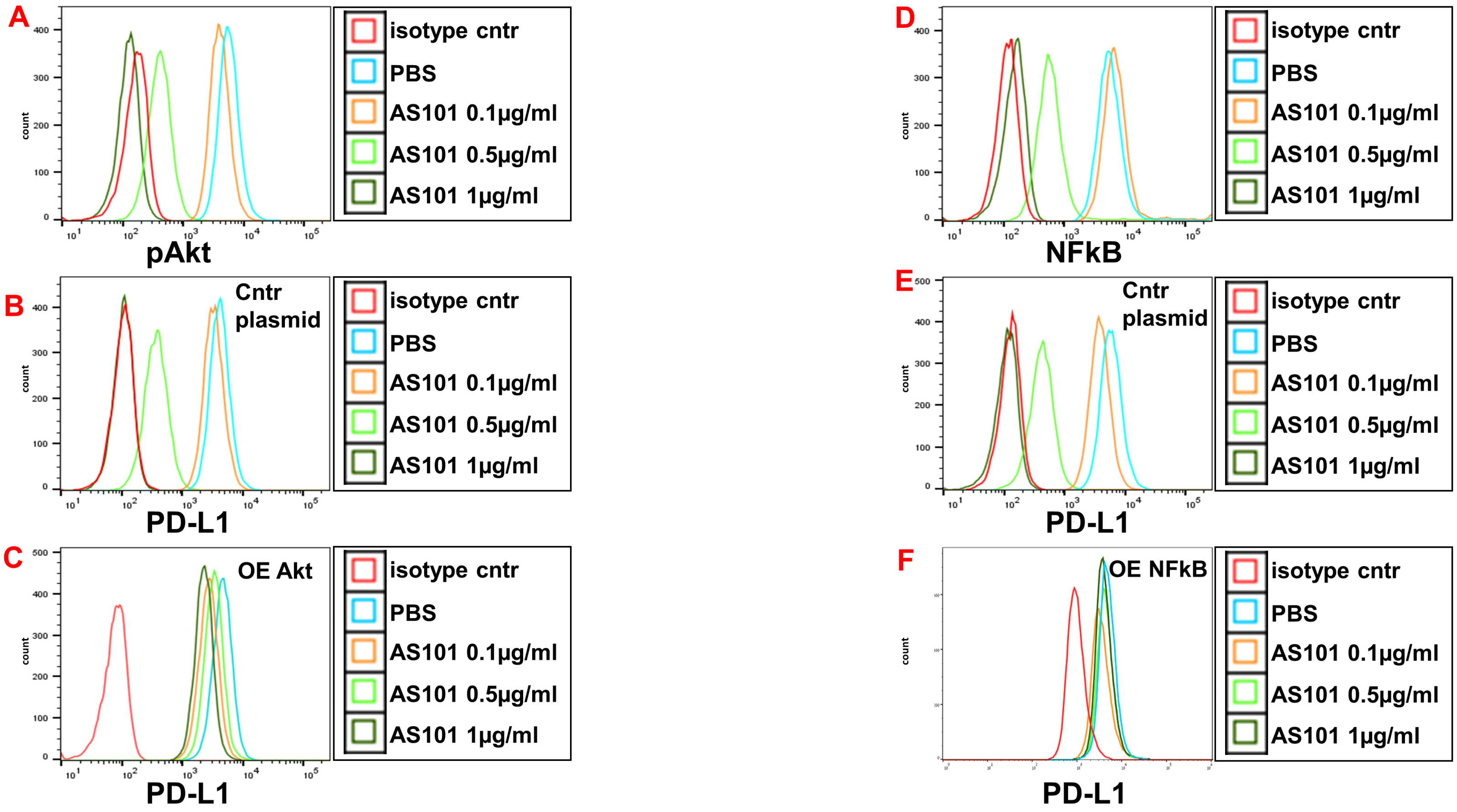

A549
A
pAkt
isotype cntr
PBS
AS101 0.1µg/ml
AS101 0.5µg/ml
AS101 1µg/ml
B
Cntr plasmid
PD-L1
C
OE Akt
PD-L1
A549
D
NFkB
E
Cntr plasmid
PD-L1
F
OE NFkB
PD-L1
count

Fig S4

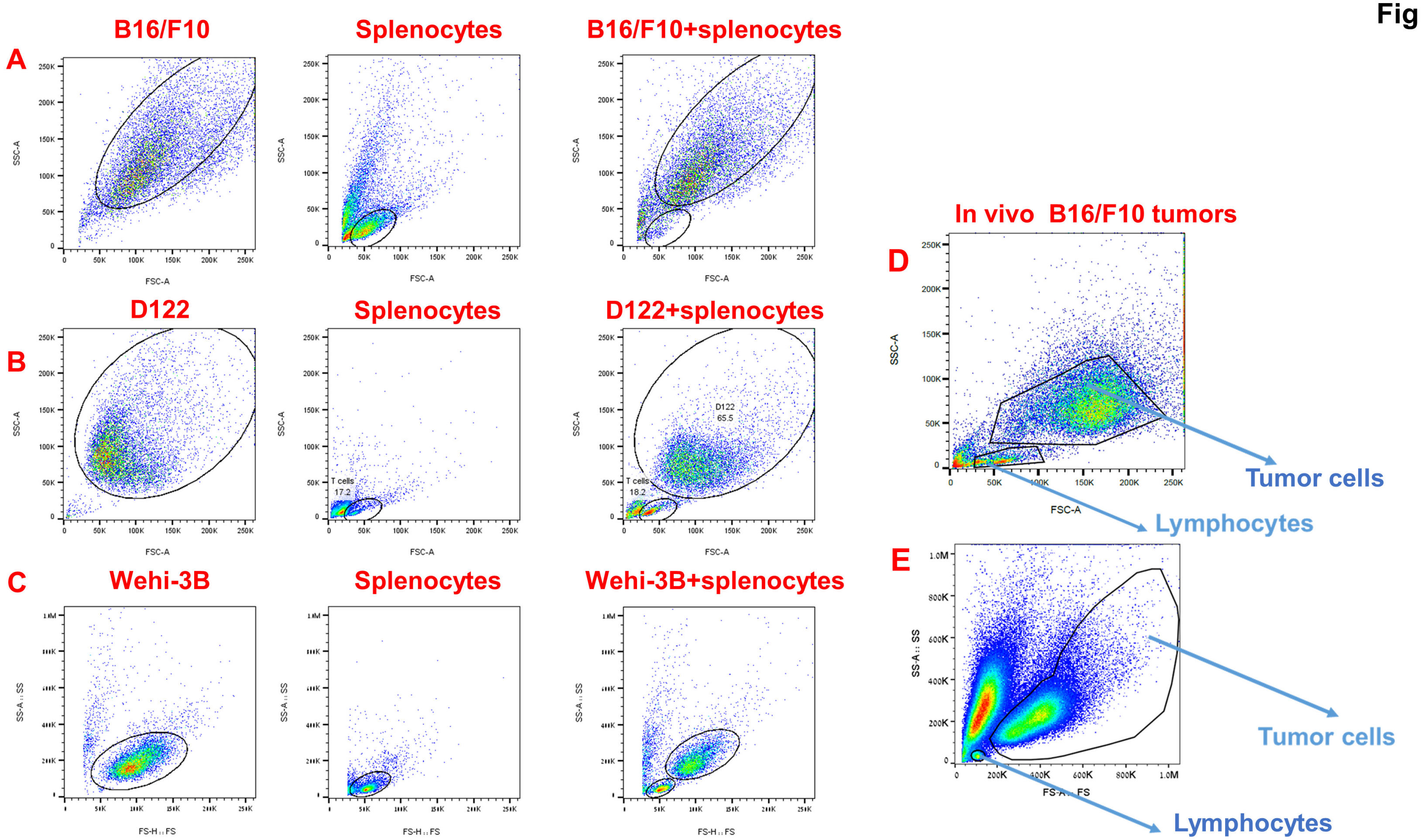
A
B16/F10
Splenocytes
B16/F10+splenocytes
B
D122
Splenocytes
D122+splenocytes
D122
65.5
T cells
17.2
T cells
18.2
C
Wehi-3B
Splenocytes
Wehi-3B+splenocytes
SSC-A
FSC-A
SS-A :: SS
FS-H :: FS
D
In vivo B16/F10 tumors
Tumor cells
Lymphocytes
E
Tumor cells
Lymphocytes
FS-A :: FS

Fig. S5

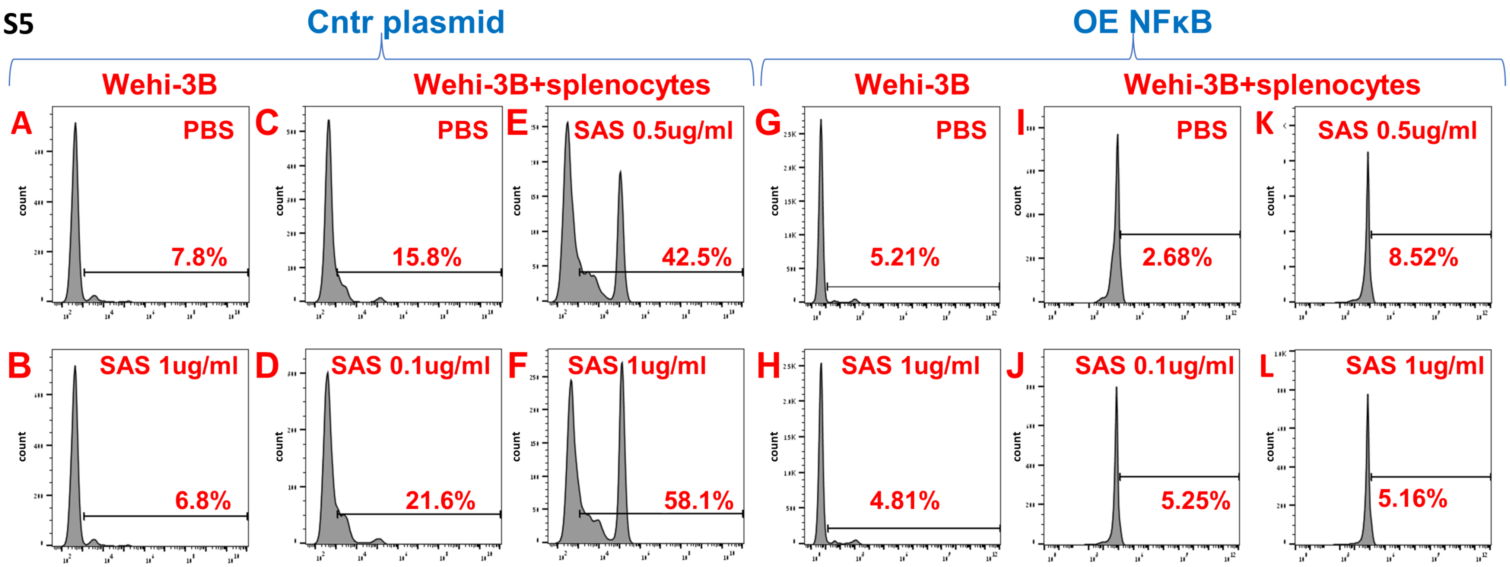

Cntr plasmid
OE NFκB
Wehi-3B
Wehi-3B+splenocytes
Wehi-3B
Wehi-3B+splenocytes
A
PBS
7.8%
C
PBS
15.8%
E
SAS 0.5ug/ml
42.5%
G
PBS
5.21%
I
PBS
2.68%
K
SAS 0.5ug/ml
8.52%
B
SAS 1ug/ml
6.8%
D
SAS 0.1ug/ml
21.6%
F
SAS 1ug/ml
58.1%
H
SAS 1ug/ml
4.81%
J
SAS 0.1ug/ml
5.25%
L
SAS 1ug/ml
5.16%
count

Fig. S6

a

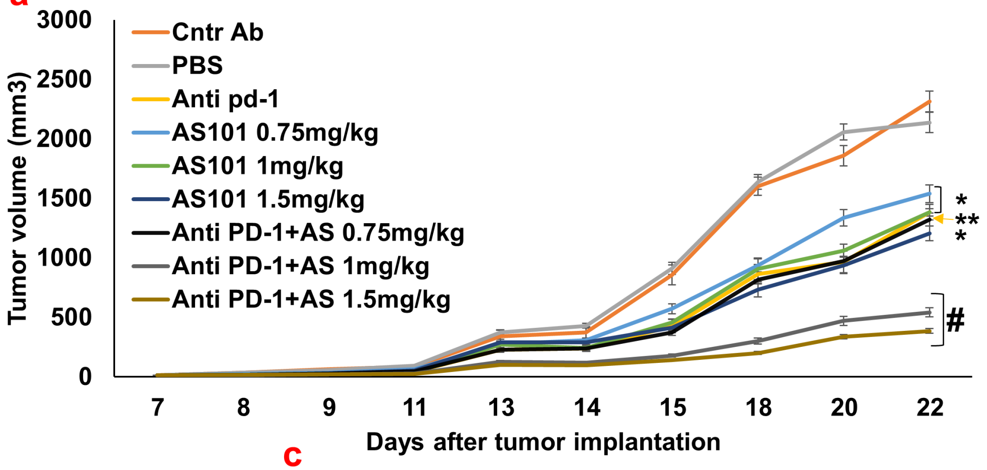

b

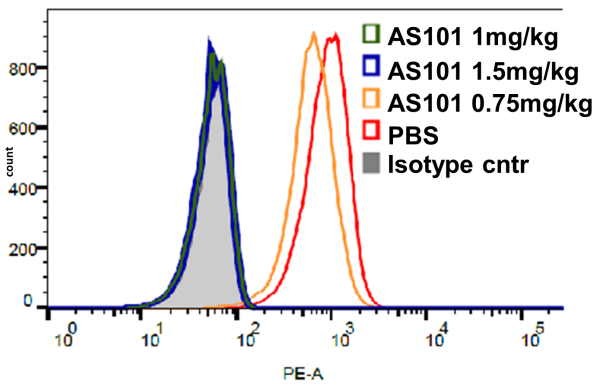

c

Sample	% CD8+ penetration	
PBS	2.40±0.12	
Cntr Ab	2.35±0.10	
AS101 (0.75mg/kg)	2.93±0.08	
AS101 (1mg/kg)	4.79±0.10	*p<0.01 vs PBS
AS101(1.5mg/kg)	4.98±0.07	*p<0.01 vs PBS
aPD-1	4.56± 0.08	*p<0.01 vs cntr Ab
aPD-1+AS101 (0.75mg/kg)	4.87±0.16	
aPD-1+AS101 (1mg/kg)	8.74±0.20	*p p<0.01 vs aPD-1 or AS101
aPD-1+AS101 (1.5mg/kg)	9.43±0.12	*p<0.01 vs aPD-1 or AS101
